# Assembly and biological functions of metal-biomolecule network nanoparticles formed by metal-phosphonate coordination

**DOI:** 10.1126/sciadv.ads9542

**Published:** 2024-12-13

**Authors:** Wanjun Xu, Zhixing Lin, Chan-Jin Kim, Zhaoran Wang, Tianzheng Wang, Christina Cortez-Jugo, Frank Caruso

**Affiliations:** Department of Chemical Engineering, The University of Melbourne, Parkville, Victoria 3010, Australia.

## Abstract

Metal-organic networks have attracted widespread interest owing to their hybrid physicochemical properties. Natural biomolecules represent attractive building blocks for these materials because of their inherent biological function and high biocompatibility; however, assembling them into coordination network materials, especially nanoparticles (NPs), is challenging. Herein, we exploit the coordination between metal ions and phosphonate groups, which are present in many biomolecules, to form metal-biomolecule network (MBN) NPs in aqueous solution at room temperature. Various phosphonate-containing biomolecules, including plant phytate, DNA, and proteins, were used to assemble MBN NPs with tunable physicochemical properties (e.g., size). In addition to excellent biocompatibility and high cargo-loading efficiency (>95%), these two-component MBN NPs have various biological functionalities, including endosomal escape, immune regulation, and molecular recognition, thus offering advantages over nonbiomolecular-based coordination materials. This work expands our understanding of metal-organic chemistry with the emerging class of metal-biomolecule systems and provides a pathway for incorporating biofunctionalities into advanced coordination materials for diverse fields.

## Teaser

Phosphonate-containing biomolecules, e.g., DNA, coordinate with metal ions to form nanoparticles for biological applications.

## INTRODUCTION

Metal-organic materials have received substantial interest over the past three decades owing to their tunable composition and hybrid physicochemical properties (*1*–*4*). The types of organic ligands and their coordination bonding with metal ions play an important role in determining their properties and applications (*5*–*8*). For instance, metal-phenolic networks (MPNs), composed of polyphenols and metal ions, are adhesive supramolecular materials that can coat various substrates within minutes due to diverse interactions (e.g., hydrogen bonding and π interactions) formed between the substrates and the catechol/gallol groups of the polyphenols (*9*–*12*). Extensive efforts have been made to engineer the coordination chemistry of MPNs to achieve advanced functionalities (e.g., glucose-responsive, light-responsive, and photothermal properties) for specific applications (*13*–*16*). However, the properties and functionalities of MPNs are limited by metal-catechol coordination, restricting their further exploration across different fields (*17*, *18*). Exploring coordination beyond metal-catechol bonds is expected to provide additional versatility and functionality to metal-organic materials, enabling broader material design and applications.

**Fig. 1.**
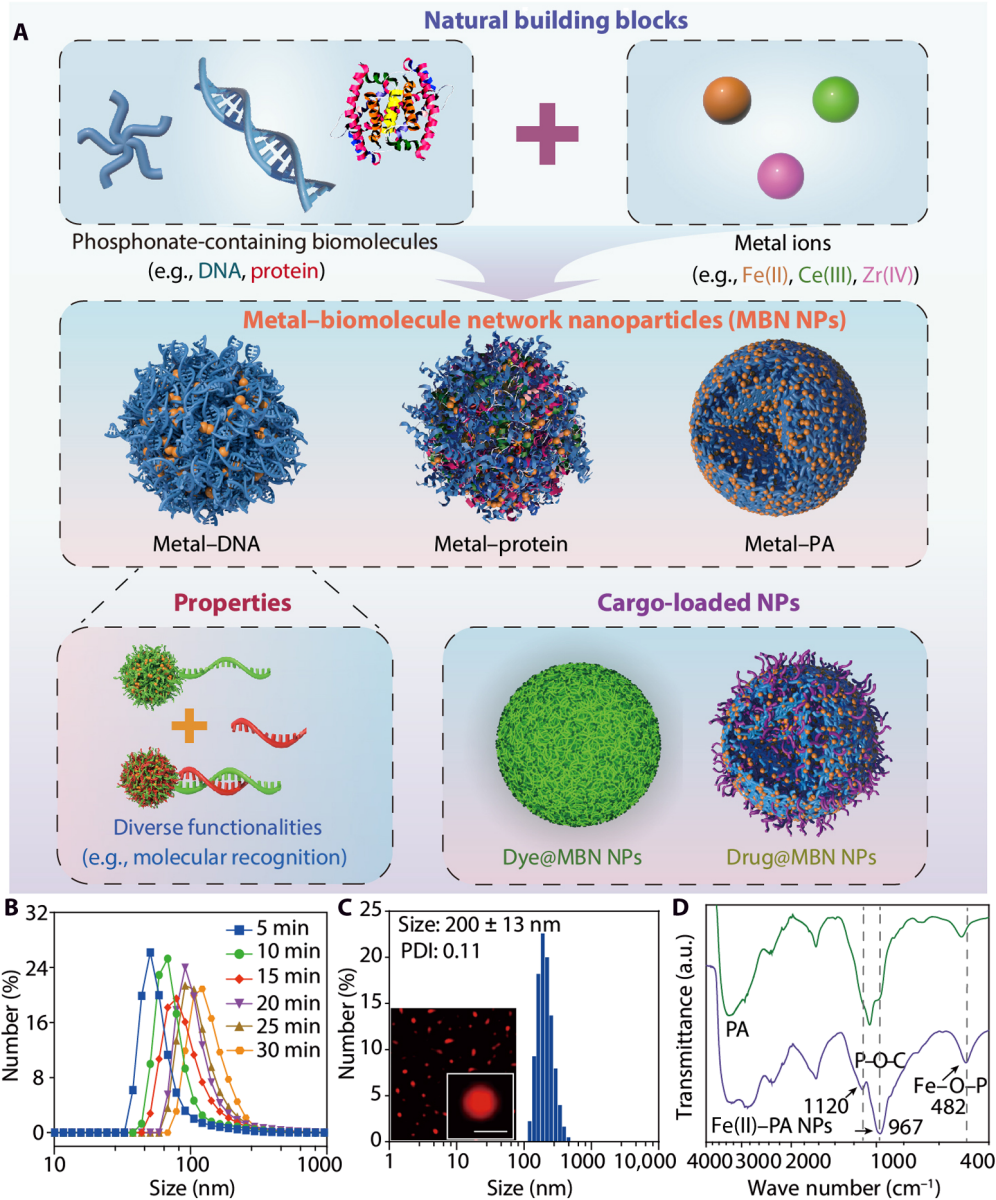
Assembly of MBN NPs. (**A**) Schematic of the assembly of MBN NPs with tunable compositions (e.g., protein and DNA) and functionalities (e.g., molecular recognition) and the assembly of cargo-loaded MBN NPs. (**B**) Time-dependent DLS data, showing the growth in size of MBN (Fe(II)-PA) NPs. (**C**) Size distribution and super-resolution lattice SIM images (inset) of RhB-labeled MBN (Fe(II)-PA) NPs. Scale bar, 200 nm. PDI, Polydispersity index. (**D**) FTIR spectra of PA and Fe(II)-PA NPs. a.u., arbitrary units.

Phosphonate-containing biomolecules, including DNA and adenosine triphosphate (ATP), are ubiquitous in living organisms and have specific physicochemical properties and biological functions (*19*–*21*). Moreover, the phosphonate groups in these biomolecules can coordinate with metal ions to form functional complexes for various biological processes such as energy transfer, signal transduction, and biosynthesis (*22*–*26*). However, engineering these functional complexes into coordination network materials, in particular nanoparticles (NPs), is challenging and largely unexplored despite the potential of metal-organic NPs in biomedical applications owing to their high surface areas, tunable sizes, and diverse biofunctionalities (*27*–*30*). Herein, we report a library of metal-biomolecule network (MBN) NPs with customized properties by exploiting the coordination between metal ions and phosphonate-containing biomolecules (Fig. 1A). The mechanisms underlying the formation of the MBN NPs were examined, and the physicochemical and biological properties of MBN NPs (e.g., size and molecular recognition) were tuned by varying the type of biomolecule, the valence state of the metal ions, the metal-to-ligand ratio, the precursor concentration, and the reaction time. In addition, various cargos, including fluorescent dyes, anticancer drugs, and proteins, were readily incorporated into the MBN NPs with high loading efficiencies to achieve customized functionalities. Moreover, the high biocompatibility, endosomal escape capability for intracellular cargo, and diverse biofunctionality of MBN NPs make them promising candidates for a range of biomedical applications. For instance, the metal-DNA NPs were examined as an immunotherapeutic adjuvant and exhibited enhanced immune responses compared to naked DNA, highlighting the potential of MBN NPs for vaccination. This study expands the realm of metal-organic materials and provides an avenue for exploiting coordination chemistry and introducing biofunctionality in preparing advanced hybrid metal-organic materials for diverse applications.

## RESULTS

### Assembly of MBN NPs

We used a one-pot strategy to fabricate MBN NPs by mixing metal ions and phosphonate-containing biomolecules in an aqueous buffer solution [e.g., phosphate buffer (PB)] at ambient temperature (Fig. 1A). Fe(II) and phytic acid (PA), a natural plant–derived biomolecule containing phosphonate groups only, were chosen as a model metal ion and a model ligand, respectively (fig. S1). PA was selected to simplify the system to examine the effect of metal-phosphonate coordination on the NP formation and properties. We note that additional functional groups (e.g., hydroxyl or amine groups) may also be present in phosphonate-containing biomolecules, which could influence NP formation. The formation of the MBN NPs (i.e., Fe(II)-PA NPs) was monitored by time-dependent dynamic light scattering (DLS), which showed the size of the NPs gradually increased to ~150 nm within 30 min of mixing (Fig. 1B and fig. S2). Crystalline diffraction peaks were absent from the x-ray diffraction pattern of Fe(II)-PA NPs, indicating the presence of metal-biomolecule assemblies with an amorphous structure (fig. S3). The size of the MBN NPs was also controlled by adjusting the metal-to-ligand ratio and precursor (i.e., biomolecule and metal) concentration (figs. S4 and S5). Specifically, the size of the Fe(II)-PA NPs increased from ~50 to ~200 nm when the concentrations of the components were simultaneously increased {[Fe(II)] from 0.5 to 10 mg ml^−1^ and [PA] from 1.5 to 30 mg ml^−1^} while keeping a constant Fe(II)-to-PA molar ratio of 1:1 (fig. S5). The concentration-dependent NP size changes were also supported by small-angle x-ray scattering (SAXS) measurements, wherein a higher precursor concentration resulted in a higher scattering intensity at a small scattering vector *q* with increasing NP size (fig. S6). Figure 1C shows the size distribution of rhodamine B (RhB)–labeled MBN (Fe(II)-PA) NPs, as measured by DLS, which featured a size of 200 ± 13 nm in aqueous solution; this was confirmed by super-resolution lattice structured illumination microscopy (SIM). Atomic force microscopy (AFM) analysis of the MBN (Fe(II)-PA) NPs in an air-dried state revealed a height of ~5 nm (fig. S7), attributed to the collapse of the NPs due to air drying (*29*). Fourier transform infrared (FTIR) spectroscopy analysis revealed the presence of an Fe–O–P stretching vibration band at 482 cm^−1^, as well as the anti-symmetrical and symmetrical frequencies of P–O–C groups with a shift to lower (from 996 to 967 cm^−1^) or higher (from 1060 to 1120 cm^−1^) frequencies, suggesting coordination between PA and metal ions within the MBN NPs (Fig. 1D) (*31*, *32*).

**Fig. 2.**
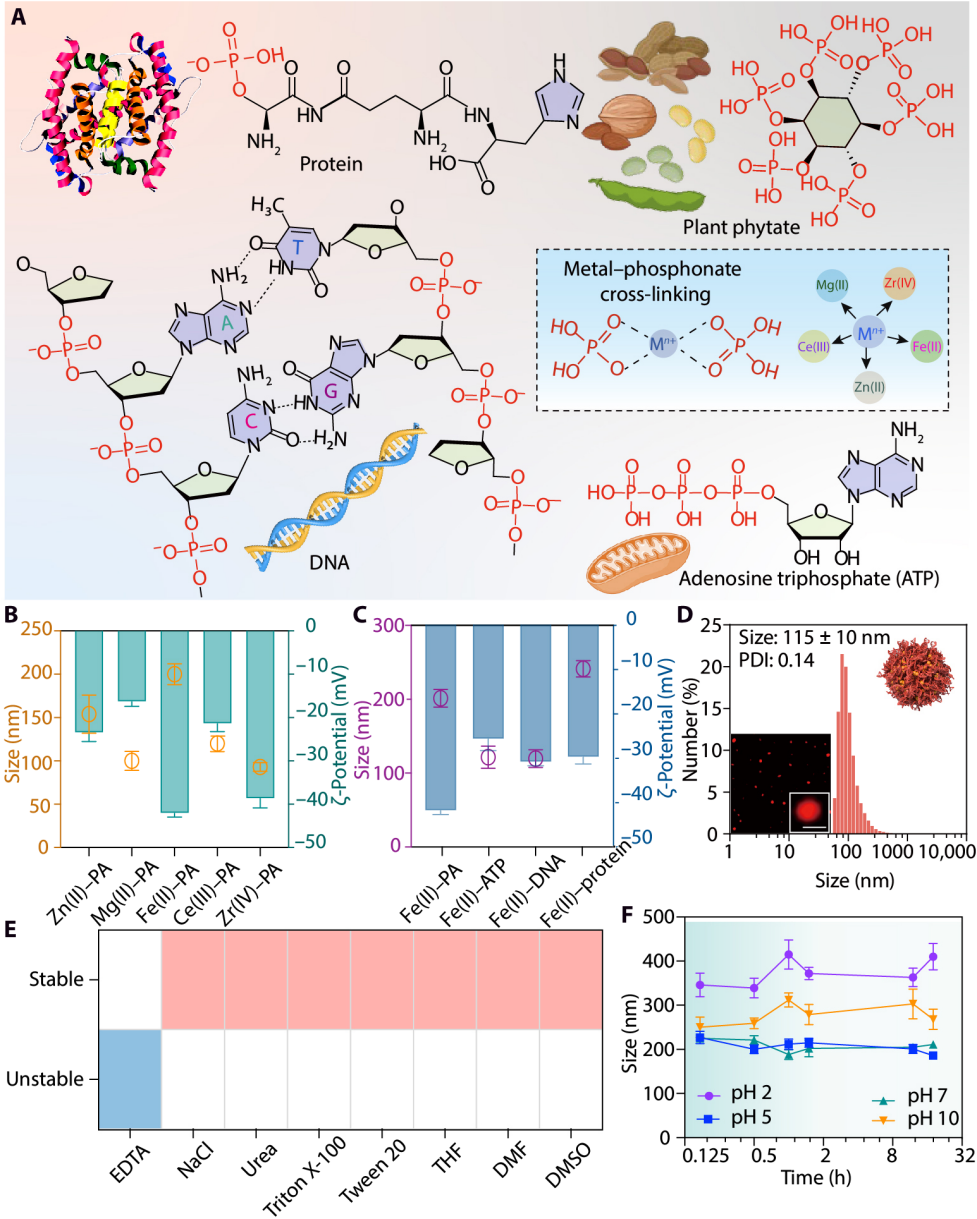
Tuning the versatility and stability of MBN NPs. (**A**) Chemical structures of phosphonate-containing biomolecules and their possible interactions with various metal ions (dashed box). (**B** and **C**) Size, as determined by DLS, and ζ-potential values of MBN NPs assembled from PA and various metal ions (B) or Fe(II) and various phosphonate-containing biomolecules (C). Data are shown as the means ± SD (*n* = 3). (**D**) Size distribution and super-resolution lattice SIM images (inset) of Fe(II)-DNA NPs. Cy3-labeled DNA was used for imaging. Scale bar, 100 nm. (**E**) Heatmap illustrating the stability of MBN (Fe(II)-PA) NPs upon incubation in different media. (**F**) Stability of MBN [Fe(II)-PA] NPs upon incubation in different pH solutions. Data are shown as the means ± SD (*n* = 3). h, hours.

To explore the versatility of the metal-biomolecule assembly strategy, the influence of the type of phosphonate-containing biomolecule, metal ion, and buffer on the formation of NPs was assessed. Multivalent metal ions [e.g., Zn(II), Mg(II), Fe(II), Ce(III), and Zr(IV)] and biomolecular building blocks that are abundant with phosphonate groups (e.g., DNA and ATP) were selected to investigate the role of metal-phosphonate cross-linking on the formation of MBN NPs (Fig. 2A). Various metal ions coordinated with PA to form MBN NPs, which displayed a size range of 100 to 200 nm (Fig. 2B). Transmission electron microscopy (TEM) revealed the collapsed nature of the NPs following air drying (fig. S8). Ultraviolet-visible (UV-vis) spectroscopy of the MBN NPs indicated the presence of metal-PA coordination from the shift of the PA absorbance band (fig. S9). In addition, the use of other biomolecules with distinctly different structures from PA (e.g., DNA, protein, and ATP) led to the formation of MBN NPs with comparable size (100 to 250 nm) and ζ-potentials ranging from ~−25 to −45 mV (Fig. 2C). Cyanine 3 (Cy3)–labeled DNA was used to visualize the assembly of metal ions and DNA—monodisperse Fe(II)-DNA NPs were obtained (Fig. 2D). The coordination between metal ions and phosphate groups of DNA in Fe(II)-DNA NPs is indicated by the presence of an Fe–O–P stretching vibration band at 546 cm^−1^ and a shift of the symmetrical stretching vibration peak of P–O groups (from 1084 to 1008 cm^−1^) in the FTIR spectra (fig. S10) (*33*, *34*). A range of physiologically relevant buffer solutions was used to investigate the robustness of the metal-phosphonate assembly. Fe(II)-PA NPs and Fe(II)-ATP NPs were formed when either 3-morpholinopropane-1-sulfonic acid (MOPS), citrate buffer, or PB was used. In contrast, the formation of Fe(II)-DNA NPs only occurred in PB and imidazole (fig. S11). The stabilizing interactions of MBN [Fe(II)-PA] NPs were further examined by incubating the NPs in various competitor solvents. MBN NPs disassembled after incubation with EDTA, confirming the presence of metal coordination. In contrast, the MBN NPs remained stable after incubation with ionic competitors (e.g., NaCl), hydrogen bond competitors (e.g., urea), hydrophobic competitors (e.g., Triton X-100 and Tween 20), or π competitors [e.g., *N*,*N*′-dimethylformamide (DMF) and tetrahydrofuran (THF)] (Fig. 2E). Note that the stability of MBN NPs is evaluated in terms of their degree of nondisassembly, as indicated by particle size measurements, unless otherwise specified. In addition, given the vast number of phosphonate-containing biomolecules in nature, with various structures and additional functional groups (e.g., hydroxyl and amine groups), other interactions (e.g., hydrogen bonding) may be present in the MBN NP systems, depending on the biomolecule involved. The MBN NPs showed high and prolonged stability in biological media [i.e., Dulbecco’s phosphate-buffered saline (DPBS), human plasma, and Dulbecco’s modified Eagle’s medium (DMEM) with 10% fetal bovine serum (FBS)], compared to their stability in EDTA, which is used as the control for disassembly (fig. S12). Note that the MBN NPs were stable over a wide pH range, even under highly acidic (e.g., pH 2 and pH 3) or highly alkaline (e.g., pH 10 and pH 12) conditions (Fig. 2F and figs. S13 and S14), likely due to the robust and diverse coordination states (e.g., monovalent to tetravalent) formed between metal ions and the strongly electron-donating phosphonate groups (*35*).

### Encapsulation of diverse cargos

**Fig. 3.**
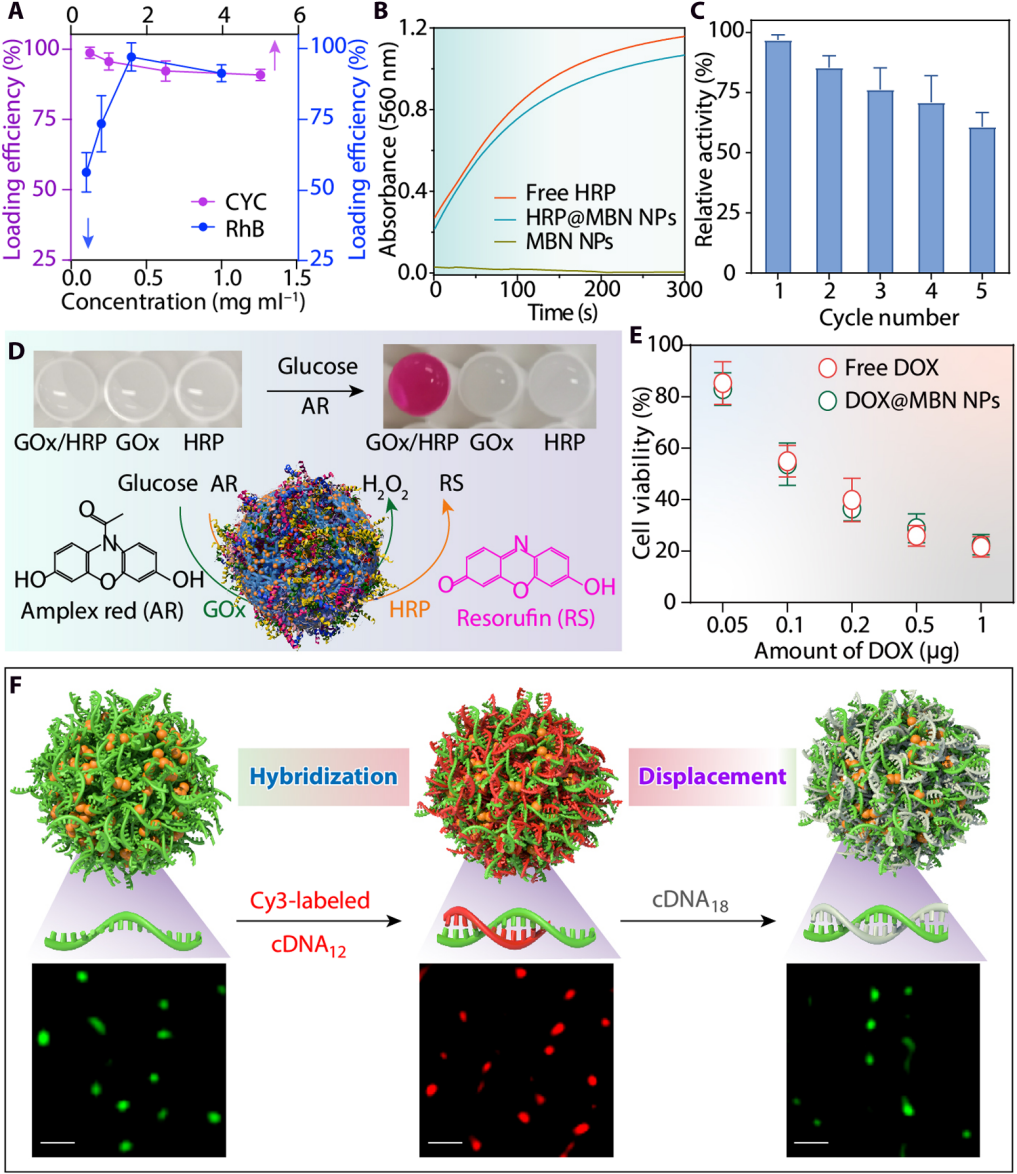
Loading capacity of MBN NPs and performance of cargo-loaded MBN NPs. (**A**) Loading efficiencies of RhB and CYC in MBN [Fe(II)-PA] NPs. Data are shown as the means ± SD (*n* = 3). (**B**) Time-dependent absorbance changes upon oxidation of amplex red by different catalytic systems: free HRP, MBN NPs, and HRP@MBN [Fe(II)-PA] NPs. (**C**) Relative catalytic activity of HRP@MBN [Fe(II)-PA] NPs as a function of cycle number. (**D**) Schematic of the enzymatic cascade reactions of GOx/HRP@MBN NPs, wherein GOx catalyzes the oxidation of glucose to H_2_O_2_ and HRP catalyzes the oxidation of amplex red (AR) to resorufin (RS). Inset: Photographs showing the resulting color changes using different single-component and multicomponent NP systems. (**E**) Viability of HeLa cells after incubation with free DOX or DOX@MBN NPs at different drug dosages. Data are shown as the means ± SD (*n* = 5). (**F**) Demonstration of the functionality of Fe(II)-DNA NPs including hybridization and displacement using complementary oligonucleotides cDNA_12_(Cy3) and cDNA_18_. Super-resolution lattice SIM images of the corresponding Fe(II)-DNA NPs are shown. Scale bars, 500 nm.

The MBN NPs were then explored for loading functional cargos, including nonphosphonate-containing small molecules and proteins. To characterize the loading capacity of MBN [Fe(II)-PA] NPs, RhB and cytochrome c (CYC) were selected as model small molecule and protein cargos, respectively. The cargo-loaded MBN NPs were also prepared using a one-pot strategy, wherein the cargos were incorporated during NP assembly. UV-vis spectroscopy confirmed the successful incorporation of RhB and CYC into the MBN NPs (fig. S15), with loading efficiencies of 97 and 98%, respectively. These results suggest the high loading efficiency of MBN NPs for small molecules and biomacromolecules (Fig. 3A and fig. S16). The cargo-loaded MBN NPs were then explored for various applications, including biocatalysis and drug delivery. For example, horseradish peroxidase (HRP), a common catalytic enzyme that is difficult to recycle owing to its relatively small size, was incorporated into the MBN NPs. The resulting NPs showed comparable catalytic activity to free HRP (Fig. 3B). The recyclability of HRP@MBN NPs was examined, and 60% of the initial HRP activity was retained after five cycles of catalysis and collection (Fig. 3C). Moreover, multicomponent systems composed of glucose oxidase (GOx) and HRP were engineered to enable enzymatic cascade reactions (Fig. 3D). As designed, GOx within the MBN NPs can catalyze the oxidation of glucose to gluconic acid and H_2_O_2_, and the produced H_2_O_2_ is then used for the HRP-mediated oxidation of amplex red in the enzymatic cascade (fig. S17) (*36*). In addition, a small molecule drug, doxorubicin (DOX), was incorporated into the MBN [Fe(II)-PA] NPs with a high loading efficiency of 98% and exhibited comparable anticancer activity to that of free DOX (Fig. 3E and figs. S18 and S19). These data show that the metal-biomolecule assembly strategy can not only incorporate phosphonate-containing biomolecules into NPs but also further load diverse cargos for specific applications.

### Molecular recognition

Oligonucleotides, such as DNA and RNA, with a phosphonate backbone (Fig. 2A), have molecular recognition properties that are essential for various biological and biomedical applications, including the delivery of genetic information, encoding instructions for protein synthesis, and biosensing (*37*–*39*). To investigate the molecular recognition properties of MBN NPs, single-stranded DNA conjugated with a fluorescein amidite (FAM) dye and Fe(II) were selected as a model ligand and metal for fabricating metal-DNA NPs. Sequence-specific binding interaction of the Fe(II)-DNA NPs was determined based on Förster resonance energy transfer between DNA (FAM) in the NPs and a Cy3-labeled cDNA with a nucleotide length of 12 (cDNA_12_; table S1). Complementary binding or hybridization resulted in a decrease in the fluorescence intensity of FAM in the DNA strands, as observed by fluorescence spectroscopy (fig. S20). Molecular recognition between Fe(II)-DNA NPs and cDNA is dynamic and can be further displaced by a third cDNA (Fig. 3F and table S1). For example, upon the further addition of a cDNA with a longer nucleotide length (cDNA_18_), which has a larger hybridization binding energy than cDNA_12_ (table S2), displacement of cDNA_12_ occurred, and the green fluorescence of the NPs was recovered (Fig. 3F and fig. S21). The process of hybridization and displacement was accompanied by a slight change in the ζ-potential of MBN NPs (fig. S21B). The NPs remained stable in the reaction buffer, as suggested by the negligible change in particle size (fig. S21A), indicating that the engineered metal-nucleotide NPs have potential as DNA-based sensors with high sensitivity and stability (*40*, *41*).

### Broad-spectrum biological applications

**Fig. 4.**
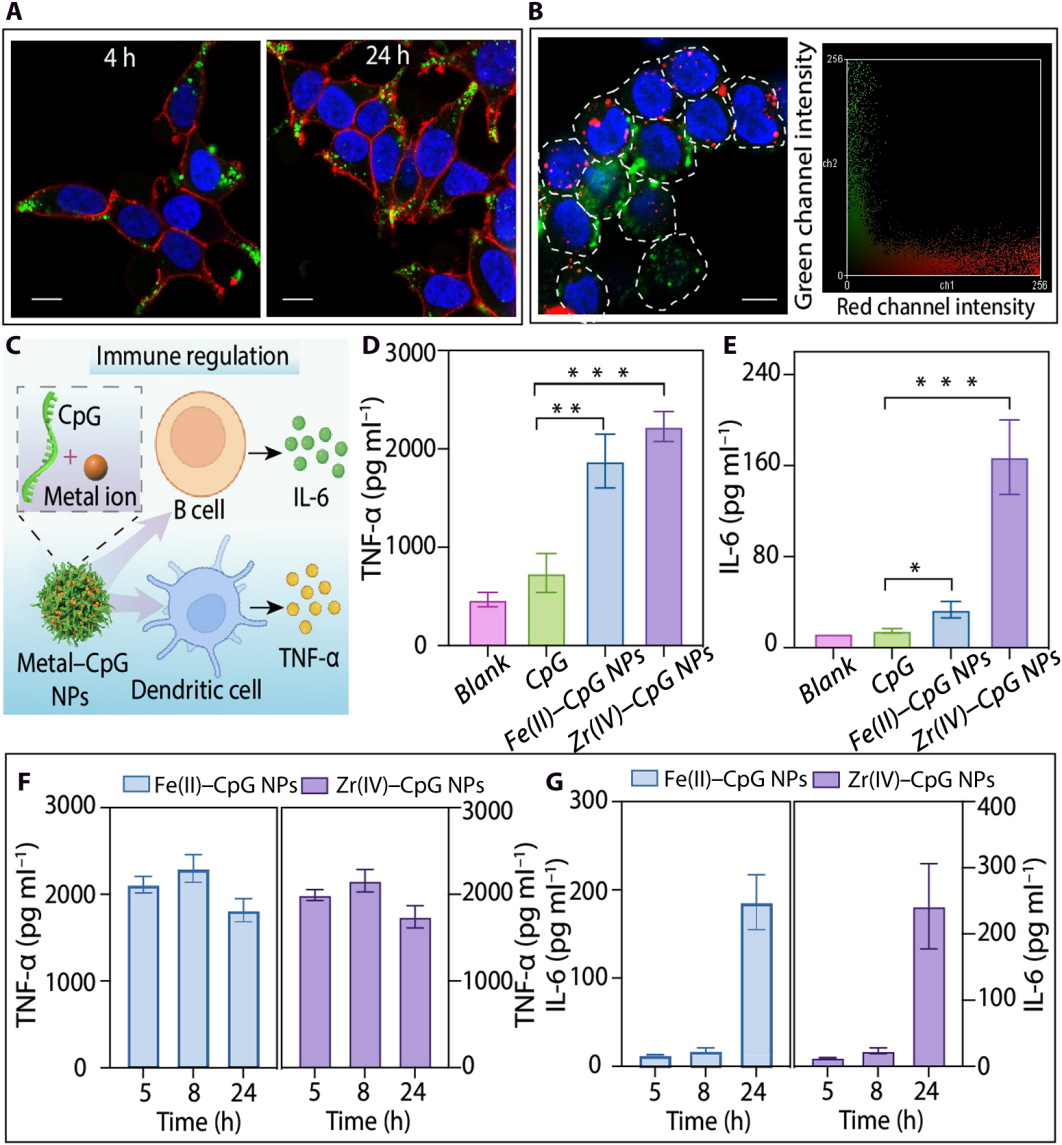
Biological functionalities and applications of MBN NPs. (**A**) CLSM images of cells after incubation with FITC-BSA@MBN [Fe(II)-PA] NPs for 4 and 24 hours. Green, FITC-BSA@MBN NPs; blue, cell nuclei; red, cell membrane. Scale bars, 10 μm. (**B**) CLSM image and associated color scatterplot showing the intracellular delivery of BSA-loaded MBN NPs in HEK 293T cells after incubation for 8 hours. Green, FITC-BSA@MBN NPs; blue, nuclei; red, endosomes and lysosomes. Scale bar, 10 μm. (**C**) Schematic of immune response induced by metal-CpG NPs via cytokine secretion. (**D** and **E**) ELISA measuring the release of TNF-α (D) and IL-6 (E) from RAW264.7 macrophage cells treated with CpG or metal-CpG NPs for 24 hours (the concentration of the CpG motif in all samples was 200 nM). Data are shown as the means ± SD (*n* = 5). *t* test: *P < 0.05, **P < 0.01, and ***P < 0.001. (**F** and **G**) Level of secretion of TNF-α and IL-6 from RAW264.7 cells incubated with Fe(II)-CpG NPs or Zr(IV)-CpG NPs [(CpG) = 260 nM] for 5, 8, and 24 hours. Data are shown as the means ± SD (*n* = 5). h, hours.

The distinct properties and advantages of MBN NPs, derived from biomolecules and coordination networks, for various biological applications were then examined. The MBN NPs are highly biocompatible—as shown in fig. S22, Fe(II)-PA and Fe(II)-DNA NPs displayed negligible cytotoxicity at increasing NP-to-cell ratios of up to 200,000:1. The coordination networks of MBN NPs facilitate efficient intracellular delivery of functional cargos. For instance, the cellular internalization and association of MBN NPs with human embryonic kidney (HEK) 293T cells were visualized using a fluorescently labeled cargo [i.e., fluorescein isothiocyanate–labeled bovine serum albumin (FITC-BSA)]. As shown in Fig. 4A, FITC-BSA@MBN [Fe(II)-PA] NPs were internalized by the cells, with the percentage of positive cells (FITC-BSA@MBN NPs associated) reaching 91% after 4 hours of incubation (figs. S23 and S24). Notably, the internalized FITC-BSA@MBN NPs exhibited efficient endosomal escape, indicated by the limited NP and lysosome colocalization as quantified by the Pearson’s correlation coefficient (0.39) (Fig. 4B and fig. S25). This is likely due to the “proton-sponge effect” from the buffering capacity of metal-organic coordination networks (*42*, *43*).

Moreover, diverse functional biomolecules can be incorporated into MBN NPs to achieve desired biological properties for targeting biological applications. Cytosine-phosphorothioate-guanosine (CpG), an immunoregulatory nucleic acid that acts as an adjuvant to strengthen the immune response (*44*, *45*), was selected as the organic ligand to fabricate MBN NPs (Fig. 4C). Naked CpG is often easily degraded by nucleases; however, the formation of MBN NPs can stabilize CpG and enable intracellular delivery of immunostimulatory CpG motifs without using toxic carriers. Upon activation of the CpG motifs, several proinflammatory cytokines [e.g., tumor necrosis factor–α (TNF-α) and interleukin-6 (IL-6)] are typically secreted from immune cells and amplify the immune responses to immunotherapies (*46*). Incubation with naked CpG induced limited secretion of TNF-α and IL-6 in RAW264.7 cells. In contrast, incubation with Fe(II)-CpG NPs or Zr(IV)-CpG NPs induced high levels of TNF-α and IL-6 in RAW264.7 cells (Fig. 4, D and E), which is attributed to the protection of CpG from nuclease degradation and efficient cellular uptake of the metal-CpG NPs. Furthermore, the metal-CpG NPs stimulated the secretion of TNF-α and IL-6 in a time-dependent manner and reached a maximum stimulatory effect and likely a saturated plateau (*47*, *48*) after incubation for 24 hours (Fig. 4, F and G). Moreover, small interfering RNA (siRNA), a promising therapeutic agent for treating diseases involving aberrant protein production (*49*), was selected as a model biomolecule to demonstrate the versatile biological potential of MBN NPs. Specifically, MBN NPs fabricated with enhanced green fluorescent protein–targeted siRNA (siEGFP) achieved comparable gene knockdown performance [~50% knockdown for Zr(IV)-siEGFP NPs] to that of commercial transfection agent Lipofectamine RNAiMAX and effectively silenced EGFP expression in HeLa-EGFP cells (figs. S26 and S27). Collectively, these results suggest that MBN NPs are promising platforms for biological applications due to their high biocompatibility, efficient endosomal escape for intracellular cargo delivery, and diverse biofunctionalities.

### Modular assembly of supraparticles using MBN NPs

**Fig. 5.**
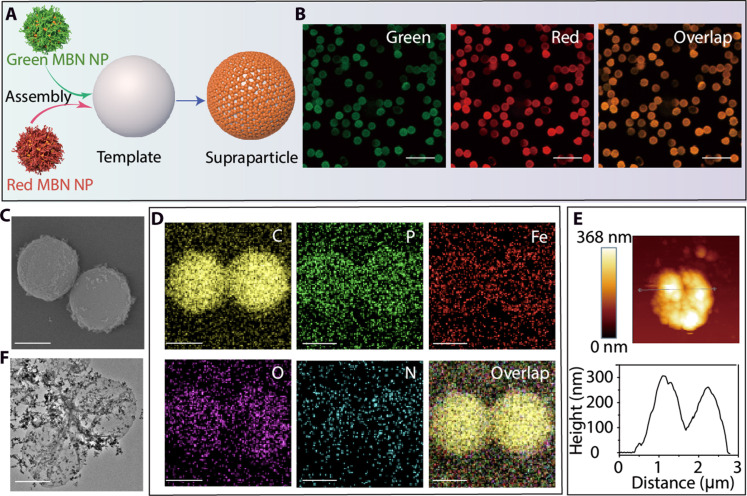
Assembly and characterization of MBN supraparticles. (**A**) Schematic of the formation of superstructures through DNA-based MBN particles via templated assembly. (**B**) Super-resolution lattice SIM images of MBN supraparticles under green and red channels. Scale bars, 5 μm. (**C** and **D**) SEM image and EDX elemental mapping of MBN supraparticles. Scale bars, 1 μm. (**E**) AFM image and corresponding thickness profile of the superstructures. (**F**) TEM image of hollow supraparticles. Scale bar, 1 μm.

The assembly of biomolecules into supraparticles is of widespread interest for engineering materials with intricate structures and functionalities for various applications such as drug delivery, energy storage, and sensing (*50*, *51*). However, the formation of biomolecule-assembled superstructures often requires complicated synthesis procedures, which can limit flexibility or potentially compromise the integrity of the biomolecules (*52*, *53*). We have previously demonstrated that polyphenol-functionalized building blocks can be used for the modular assembly of superstructures with diverse compositions (*54*, *55*). Herein, we selected DNA, a biomolecule that is easily denatured, to fabricate superstructures (i.e., supraparticles) based on metal-DNA NPs. Specifically, single-stranded DNA (FAM) and its complementary sequence cDNA (Cy3) were used as biomolecular ligands to form Fe(II)-DNA NPs (green) and Fe(II)-cDNA NPs (red), respectively (Fig. 5A). The modular-assembled supraparticles were then constructed through sequence-specific interactions between DNA and cDNA of the resulting NPs on the surface of larger templates [e.g., amine-modified polystyrene (PS-NH_2_) particles with a size of 1.86 ± 0.08 μm] (Fig. 5A). We observed colocalization of the fluorescent signals (orange) of the supraparticles, which was attributed to the FAM-labeled Fe(II)-DNA NPs (green) and the Cy3-labeled Fe(II)-cDNA NPs (red) (Fig. 5B). Scanning electron microscopy (SEM) and energy-dispersive x-ray (EDX) mapping showed the morphology and the presence of O and P (from the DNA) and Fe within the supraparticles (Fig. 5, C and D and fig. S28). After template removal, the resulting hollow supraparticles in their air-dried state were observed by microscopy analyses (Fig. 5, E and F, and fig. S29), indicating that the MBN NP building blocks successfully formed three-dimensional freestanding films. With the modular, structural, and functional diversity of DNA, these DNA-based superstructures have potential for use in various sensing applications across environmental monitoring and biomedical diagnostics.

## DISCUSSION

We demonstrated a simple strategy for assembling metal-biomolecule complexes into coordination network NPs from a wide range of metal ions and phosphonate-containing biomolecules. The physicochemical and biological properties (e.g., size and cell targeting) of the MBN NPs were engineered by selecting diverse biomolecules and metal ions, as well as altering the assembly conditions (e.g., reaction time). In addition, a variety of cargos, including small molecules (e.g., anticancer drugs) and macromolecules (e.g., enzymes), were readily incorporated into the MBN NPs, achieving high loading efficiencies while preserving the functionality of the incorporated cargo. The resulting MBN NPs exhibited good compatibility, efficient intracellular cargo delivery, and biological properties (e.g., molecular recognition), indicating their potential for a wide range of biomedical applications, including drug delivery and catalysis. For example, metal-CpG NPs based on immunostimulatory CpG exhibited high immunostimulatory activity compared with naked CpG and showed promise for advanced vaccine formulation. Moreover, MBN NPs fabricated independently from single-stranded DNA and its cDNA served as building blocks to assemble supraparticle nanostructures, offering potential for sensing applications. Given the diverse biofunctionality of phosphonate-containing biomolecules and the wide range of metal ions, this work is expected to provide insights into the assembly mechanisms of MBN systems to create a library of bioactive NPs for various fields, including functional materials, environmental science, and biomedicine.

## MATERIALS AND METHODS

### Materials

PA [50% (w/w) in H_2_O], ATP, iron(II) chloride tetrahydrate (FeCl_2_·4H_2_O), zinc nitrate hexahydrate [Zn(NO_3_)_2_·6H_2_O], aluminum chloride hexahydrate (AlCl_3_·6H_2_O), magnesium chloride hexahydrate (MgCl_2_·6H_2_O), cerium(III) chloride anhydrous (CeCl_3_), zirconium(IV) chloride (ZrCl_4_), sodium hydroxide, hydrogen chloride, MOPS, imidazole, bicine, citrate, Bis-Tris, sodium phosphate dibasic heptahydrate, sodium phosphate monobasic monohydrate, RhB, urea, Triton X-100, Tween 20, NaCl, DMEM, DPBS, and FBS were purchased from Sigma-Aldrich (United States). FAM-labeled DNA and Cy3-labeled cDNA were purchased from Bioneer (Daejeon, Korea). CpG oligonucleotides were synthesized and purified by Sangon Biotech Co. Ltd. (Shanghai, China). Dimethyl sulfoxide (DMSO), EDTA, DMF, THF, 1,4-dioxane, methanol, and ethanol were purchased from Chem-Supply. PS-NH_2_ (size = 1.86 ± 0.08 μm) particles were purchased from microParticles GmbH. CYC, phosphoserine A_2_, FITC-BSA, HRP, GOx, and amplex red were purchased from Thermo Fisher Scientific (United States). HEK 293T and RAW264.7 cells were purchased from the American Type Culture Collection. DOX and 2,3-bis[2-methoxy-4-nitro-5-sulfophenyl]-2*H*-tetrazolium-5-carboxyanilide inner salt (XTT) were obtained from Life Technologies. Milli-Q water with a resistivity of 18.2 megohm·cm was obtained from a three-stage Millipore Milli-Q Plus 185 purification system (Millipore Corporation, United States).

### Characterization

SEM images and EDX mapping analyses were performed on an FEI Teneo VolumeScope at an operating voltage of 20 kV. For analysis, SEM samples were prepared by placing a drop of a sample dispersion onto a silicon wafer, followed by drying overnight. TEM imaging was performed on an FEI Tecnai F20 instrument at an operating voltage of 200 kV. AFM experiments were conducted on a JPK NanoWizard II BioAFM instrument. Confocal laser scanning microscopy (CLSM) images were taken with a Nikon A1R+ laser scanning confocal microscope (Nikon Corporation, Japan). UV-vis absorption spectra were recorded on a Specord 250 Plus spectrophotometer (Analytik Jena AG). FTIR spectroscopy analysis was conducted on a Tensor II FTIR spectrometer. The number size distribution and ζ-potentials of particles were measured via DLS on a Zetasizer Nano ZS instrument (Malvern Instrument, United Kingdom). Powder x-ray powder diffraction was performed on a Bruker D8 Advance diffractometer, and the step size was set as 0.02. SAXS data were collected at the SAXS beamlines of the Australian Synchrotron facility, part of the Australian Nuclear Science and Technology Organisation (ANSTO). Samples were examined using the small/wide-angle x-ray scattering beamline (16 keV, 7000-mm camera length using Pilatus 1M and 200K detectors, transmission mode). Scatterbrain software was used for the analysis. Super-resolution microscopy images were acquired on a Zeiss Elyra 7 lattice SIM system with a 2× PCO.EDGE 4.2 CLHS sCMOS camera. Fluorescence spectroscopy measurements were performed on an FS5 spectrofluorometer. XTT assay was conducted according to standard protocols to evaluate the cytotoxicity of the particles. The cell samples were analyzed using an Infinite M200 microplate reader (Tecan, Switzerland). Flow cytometry was performed on a BD Accuri C6 Plus flow cytometer.

### Fabrication of MBN NPs

All phosphonate-containing biomolecules and metal solutions were prepared freshly for immediate use. MBN NPs were prepared through one-pot assembly. Briefly, to prepare Fe(II)-PA NPs, FeCl_2_·4H_2_O (80 μl, 10 mg ml^−1^) and PA (88 μl, 30 mg ml^−1^) were successively added to a vial containing PB (1000 μl, 10 mM). To prepare Fe(II)-ATP, Fe(II)-protein, or Fe(II)-DNA NPs, FeCl_2_·4H_2_O (10 μl, 10 mg ml^−1^) and ATP (50 μl, 5 mg ml^−1^), phosphoserine A_2_ (10 μl, 10 mg ml^−1^), or DNA (5 μl, 20 nmol), respectively, were introduced into a vial containing PB (500 μl). The MBN NPs were stabilized by stirring the mixture for 30 min at room temperature, and the assembled NPs were washed three times via centrifugation (9000*g*, 5 min) to remove excess materials. For the fabrication of MBN NPs using other metal ions [i.e., Mg(II), Zn(II), Ce(III), and Zr(IV)], a metal-to-PA molar ratio of 1:1 was used and the same fabrication process was used. Other buffers, including MOPS, imidazole, bicine, Bis-Tris, and citrate buffer of 100 mM were used to form MBN NPs following the same procedures as described above.

### Stability of MBN NPs

To evaluate the stability of the NPs under different conditions, MBN [Fe(II)-PA] NPs were dispersed in glycine HCl (pH 2.0 or pH 3.0, 100 mM), sodium acetate (pH 4.0, pH 5.0, or pH 6.0, 100 mM), MOPS (pH 7.0, pH 8.0, or pH 9.0, 100 mM), sodium carbonate (pH 10.0, pH 11.0, or pH 12.0, 100 mM), EDTA, DPBS, human plasma, or DMEM with 10% FBS for the desired time. MBN [Fe(II)-DNA] NPs were dispersed in sodium acetate (pH 4.5, or pH 5.5, 100 mM), tris-HCl (pH 7.4, 100 mM), or sodium carbonate (pH 9.0, 100 mM) for the desired time. To determine the possible driving forces for the assembly of MBN NPs, the pellets were incubated in 100 mM urea, Tween 20, Triton X-100, NaCl, EDTA, DMF, DMSO, or THF for 12 hours. Changes in NP size were measured on a Zetasizer Nano-ZS instrument. Data are shown as the means ± SD of three independent measurements.

### Fabrication and characterization of cargo-loaded MBN NPs

Cargo-loaded MBN NPs were prepared in a one-pot process (as that used for the MBN NPs) with the inclusion of cargo proteins (i.e., FITC-BSA, CYC, HRP, and GOx), dye (i.e., RhB), or small molecule drug (i.e., DOX) at different weight ratios in deionized water or a buffer. Specifically, different concentrations of CYC (0.5, 1.0, 2.5, and 5.0 mg ml^−1^) or RhB (0.1, 0.2, 0.4, and 1.0 mg ml^−1^) were separately mixed with FeCl_2_·4H_2_O and PA and then stirred at room temperature for 30 min to load the cargo. The loaded cargo within the NPs and the unloaded cargo in the supernatant were separated by centrifugation (9000*g*, 5 min). The supernatant was collected to quantify the unloaded cargo. The absorbance of the unloaded cargo (402 nm for CYC and 554 nm for RhB) in the supernatant was measured using UV-vis spectroscopy, and the concentration was calibrated against the relevant standard curve of characteristic peaks of the cargo. The loading efficiency of cargo in MBN NPs was calculated as

$$\begin{array}{c} \text{Loading efficiency}\;\left(\%\right)\\ =\frac{\text{Total amount of cargo}-\text{Free cargo in the supernatant}}{\text{Total amount of cargo}}\;\times \;100 \end{array}$$ (1)

### Enzyme activity test

The H_2_O_2_–amplex red colorimetric reaction was used to characterize and compare the activities of free HRP and HRP@MBN [Fe(II)-PA] NPs. Briefly, MBN NPs, free HRP, or HRP@MBN NPs were mixed with a PBS solution (pH 7.4, 10 mM) containing H_2_O_2_ (20 mM) and amplex red (1 mg ml^−1^). Changes in the absorbance of the red oxidation product (resorufin) at 560 nm were monitored by UV-vis spectroscopy. The kinetic behavior of HRP was studied by monitoring the absorbance at 560 nm at 10-s intervals by UV-vis spectroscopy. Recycling experiments with HRP@MBN NPs were performed under the same conditions as described above. After one cycle, HRP@MBN NPs were retrieved by centrifugation (9000*g*, 5 min), washed with PBS, and reused in the subsequent cycle of catalysis. For the cascade reaction involving GOx/HRP@MBN NPs, glucose (10 mg ml^−1^) was used to initiate the two tandem reactions in PBS (pH 7.4, 10 mM). After completion of the reaction (20 min at 37°C), the UV-vis absorption spectra were recorded.

### In situ hybridization testing of Fe(II)-DNA MBN NPs

To perform the in situ hybridization test, FAM-labeled DNA was used as model DNA and the prepared Fe(II)-DNA NPs were immersed in a 50 mM MOPS solution (pH 7.4, 200 μl) containing 0.6 M NaCl, followed by the addition of a 20 μM Cy3-labeled cDNA_12_ solution (2 μl) to facilitate hybridization between DNA and cDNA_12_. Thereafter, the NPs were retrieved by centrifugation (9000*g*, 5 min) and resuspended in the above buffer solutions. A cDNA_18_ solution (4 μl, 20 μM) was then added to release cDNA_12_ through hybridization of DNA with cDNA_18_. The super-resolution lattice SIM images of the MBN NPs before and after hybridization were captured, and the fluorescence intensities of FAM before and after hybridization were measured by fluorescence spectroscopy.

### Fabrication of MBN supraparticles

To prepare the supraparticles, FAM-labeled DNA and Cy3-labeled cDNA_18_ were used to prepare Fe(II)-DNA NPs and Fe(II)-cDNA_18_ NPs, respectively. PS-NH_2_ particles (10 μl) were washed twice with water (2000*g*, 2 min) and redispersed in a MOPS solution (pH 7.4, 500 μl, 50 mM) containing 0.6 M NaCl, then transferred to a glass vial, and incubated with Fe(II)-DNA NPs by stirring at 1100 rpm for 2 hours. The obtained Fe(II)-DNA NP@PS-NH_2_ particles were washed three times to remove residual Fe(II)-DNA NPs from the solution, and then FeCl_2_·4H_2_O (10 μl, 10 mg ml^−1^) and Fe(II)-cDNA_18_ NPs were successively added to a vial for hybridization between DNA and cDNA_18_. The MBN supraparticles fabricated from Fe(II)-DNA NPs and Fe(II)-cDNA_18_ NPs on PS-NH_2_ particles were stabilized by stirring the mixture for 2 hours and then washed three times with water (2000*g*, 2 min) to remove the excess material. To remove the template, 1,4-dioxane (500 μl) was added dropwise to the suspension with a brief vortex. An additional volume (500 μl) of 1,4-dioxane was added to dilute the suspension, and the particles were incubated for 1 hour to dissolve the template. The resulting hollow supraparticles were washed twice with 1,4-dioxane (2000*g*, 5 min), and the remaining 1,4-dioxane was removed by subsequent washing three times with water.

### Cell viability by XTT assay

XTT-based in vitro cytotoxicity assay was performed to evaluate the cell toxicity of the MBN NPs. XTT was dissolved in complete DMEM (with 10% FBS) to prepare a solution (0.2 mg ml^−1^), and phenazine methosulfate (PMS) was dissolved in DPBS to prepare a 1 mM solution. The XTT reagent was activated by mixing with a PMS solution at a volume ratio of 400:1. HEK 293T cells were seeded on a 96-well plate at a cell density of 2 × 10^4^ cells per well. To determine the cell viability of MBN NPs prepared from different phosphonate building blocks, Fe(II)-PA and Fe(II)-DNA NPs at different NP-to-cell ratios were examined. To determine the cell viability of DOX@MBN [Fe(II)-PA] NPs, HeLa cells were seeded on a 96-well plate at a cell density of 2 × 10^4^ cells per well, and the cells were incubated with different dosages of DOX loaded in the DOX@MBN NPs, MBN NPs, and with free DOX as the control. After incubation for 24 hours at 37°C, the media in the 96-well plate was aspirated and replaced with fresh activated XTT media (100 μl). The cells were further incubated for 4 hours at 37°C, and the absorbance at 475 nm was measured relative to nontreated cells.

### Cell association of FITC-BSA@MBN NPs

HEK 293T cells were seeded in a 24-well plate at a cell density of 10 × 10^4^ cells per well and then cultured in complete media at 37°C overnight to allow cellular adhesion on substrates. After incubation, FITC-BSA@MBN [Fe(II)-PA] NPs were added to the cells at a NP-to-cell ratio of 200,000:1 and then incubated for 4, 6, 8, and 24 hours at 37°C. The percentage of cell association was calculated by measuring the fluorescence intensity of the cells. For CLSM imaging, HEK 293T cells were seeded at 3 × 10^4^ cells per well in Labtek 8-well chamber slides and incubated overnight. Then, FITC-BSA@MBN NPs were added to the cells at a NP-to-cell ratio of 200,000:1 and then incubated for 4, 6, 8, and 24 hours at 37°C. Following incubation, the cells were gently washed three times with DPBS, detached from the culture flask with trypsin, and collected by centrifugation (350*g*, 5 min). Cells were then fixed with 4% paraformaldehyde for 15 min and gently washed three times with DPBS. The membrane was stained with Alexa Fluor 549–wheat germ agglutinin (5 μg ml^−1^) for 5 min, and the nucleus was stained with Hoechst 33342 (1 μg ml^−1^) for 10 min. CLSM imaging was performed on a microscope with a 40× water immersion objective. The images were processed by the Fiji software.

### Endosomal escape of FITC-BSA@MBN NPs

HEK 293T cells were seeded into 8-well Lab-Tek chambered cover glass slides at a cell density of 3 × 10^4^ cells per well and then cultured in DMEM (400 μl) supplied with 10% FBS overnight to allow cellular adhesion on substrates. Then, FITC-BSA@MBN NPs were added to the cells at a NP-to-cell ratio of 50,000:1 and incubated for 8 hours at 37°C. After incubation, the treated cells were gently washed three times with DPBS and stained with LysoTracker Red for endosome/lysosome labeling. For nucleus staining, cells were gently washed with DPBS and incubated with Hoechst 33342 (1 μg ml^−1^) for 10 min. Live cell imaging was performed by CLSM using a 40× water immersion objective. Pearson’s correlation coefficient and color scatterplots were obtained from the WCIF ImageJ software. The experiments were repeated in triplicate, and five representative cell images (>50 cells) were used to calculate the Pearson’s correlation coefficient values.

### Cytokine assays

RAW264.7 cells were plated into 24-well culture plates at a density of 5 × 10^5^ cells per well and incubated overnight. Then, cells were incubated with free CpG, Fe(II)-CpG NPs, or Zr(IV)-CpG NPs (CpG motifs equivalent to 200 nM) at 37°C for 24 hours. For the time course experiment, Fe(II)-CpG NPs and Zr(IV)-CpG NPs with CpG motifs equivalent to 260 nM were added to the cells and incubated at 37°C for 5, 8, and 24 hours. The levels of TNF-α and IL-6 in the supernatants were determined by the enzyme-linked immunosorbent assay (ELISA) using antibody pairs specific to these cytokines and protocols recommended by the manufacturer.

### EGFP silencing

HeLa-EGFP cells were seeded on a 24-well plate at a cell density of 1 × 10^5^ cells per well overnight and then treated with PBS, siEGFP, Fe(II)-siEGFP NPs, Zr(IV)-siEGFP NPs, or RNAiMAX-transfected siEGFP for 48 hours. The concentration of siEGFP in each formulation was 2.5 μg ml^−1^. After washing with PBS, the cells were imaged using fluorescence microscopy, and the associated EGFP fluorescence intensity was analyzed by the ImageJ software.

### Minimum information reporting in bio-nano experimental literature

The studies conducted herein, including material characterization, biological characterization, and experimental details, conform to the minimum information reporting in bio-nano experimental literature (MIRIBEL) reporting standard for bio-nano research (*56*), and we include a companion checklist of these components herein.

### Statistical analysis

The data shown for each experiment were based on at least three technical replicates, as indicated in the individual figure legends. The data are presented as the means ± SDs, and *P* values were determined using a two-sided Student’s *t* test. n.s., not significant; *P < 0.05, **P < 0.01, and ***P < 0.001.

## Supplementary Materials

### This PDF file includes:

Figs. S1 to S29 Tables S1 and S2 MIRIBEL checklist References
