# Supplementary material for: Assembly and biological functions of metal-biomolecule network nanoparticles formed by metal-phosphonate coordination
Source: Sci Adv. 2024 Dec 13;10(50):eads9542. doi: 10.1126/sciadv.ads9542 (PMC11641004; doi:10.1126/sciadv.ads9542)
Supplement: Supplementary file 1 — Figs. S1 to S29 Tables S1 and S2 MIRIBEL checklist References [file sciadv.ads9542_sm.pdf]

Science Advances
AAAS

Supplementary Materials for

Assembly and biological functions of metal-biomolecule network nanoparticles formed by metal-phosphonate coordination

Wanjun Xu *et al.*

Corresponding author: Frank Caruso, fcaruso@unimelb.edu.au

Sci. Adv. **10**, eads9542 (2024)
DOI: 10.1126/sciadv.ads9542

This PDF file includes:

Figs. S1 to S29
Tables S1 and S2
MIRIBEL checklist
References

Section S1. Supporting Figures

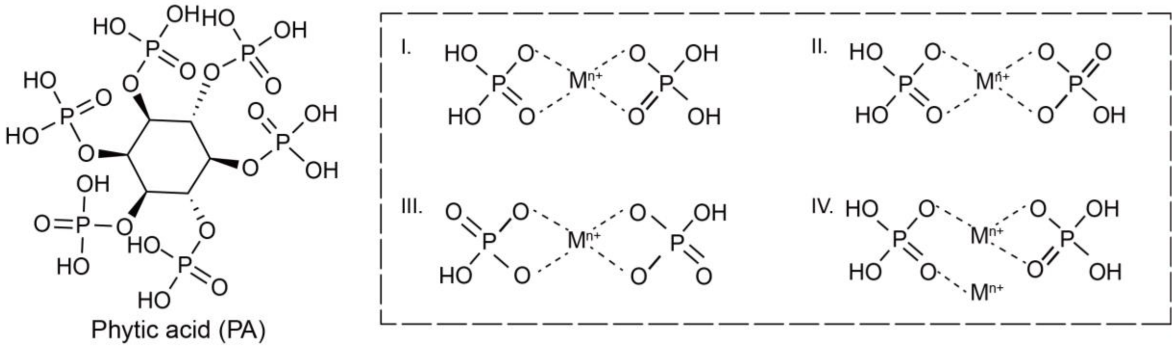

Fig. S1. Chemical structure of PA and (I─IV) possible cross-linking between metal ions and phosphonate groups (*57, 58*).

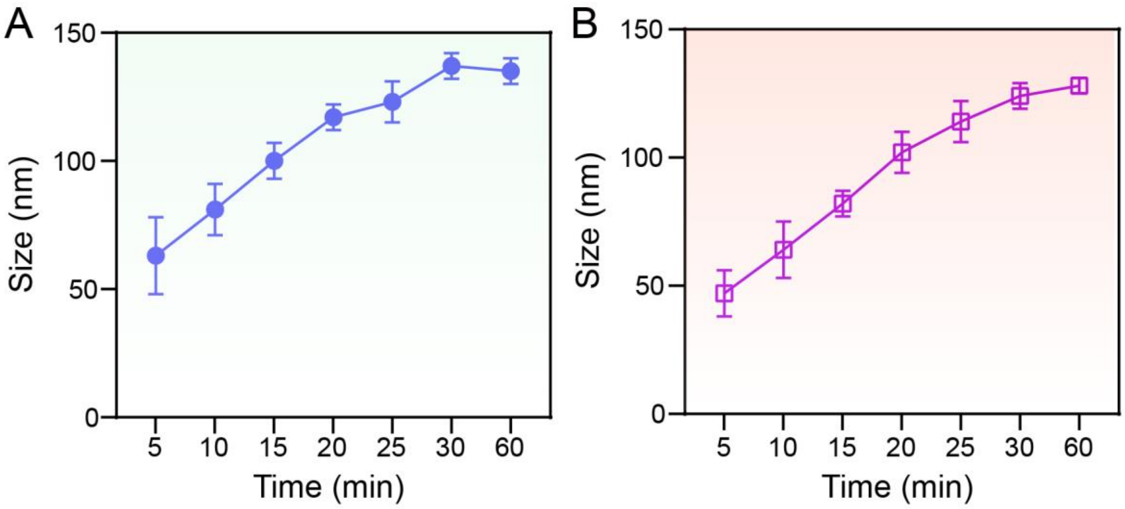

Fig. S2. Formation of MBN NPs. Size of the (A) Fe^{II}–PA and (B) Fe^{III}–PA NPs, as determined by DLS and monitored during the assembly process. Fe^{II} or Fe^{III} (5 mg mL^{-1}) and PA (15 mg mL^{-1}) were used as the NP building blocks. Data are presented as the mean ± SD, *n* = 3.

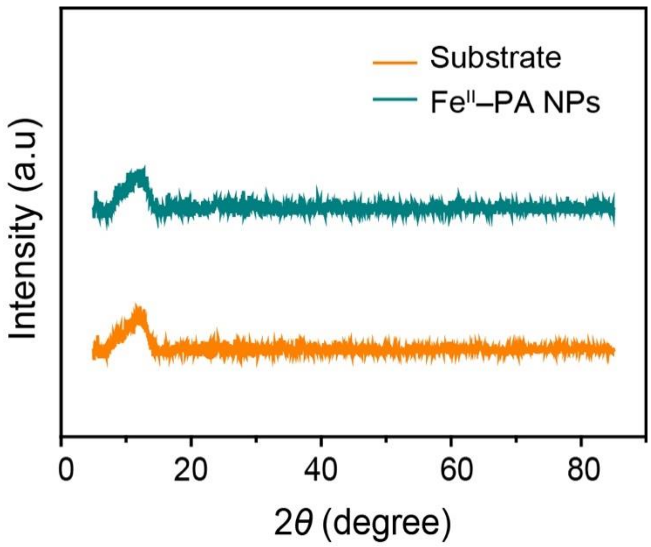

Fig. S3. XRD characterization of MBN NPs. XRD patterns of MBN (Fe^{II}–PA) NPs and substrate (i.e., quartz) used. MBN (Fe^{II}–PA) NPs were deposited onto a quartz surface for XRD measurements.

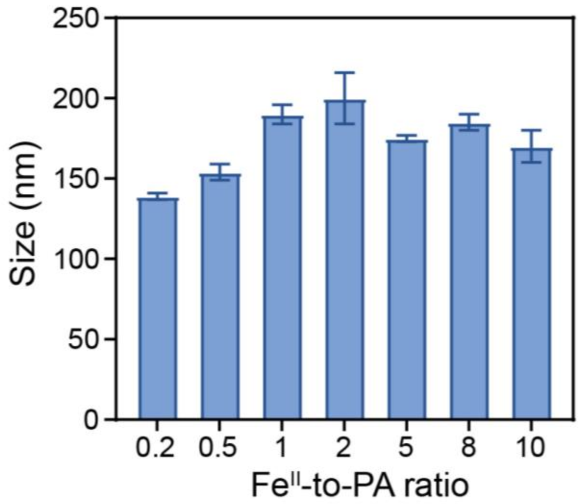

Fig. S4. Size engineering of MBN NPs through varying the metal-to-ligand ratio. Size of the MBN NPs, as determined by DLS, as a function of Fe^{II}-to-PA molar ratio. Data are presented as the mean ± SD, $n = 3$.

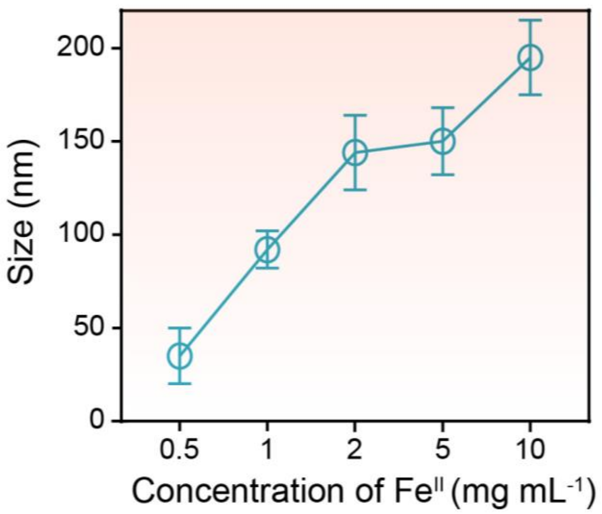

Fig. S5. Size engineering of MBN NPs through varying the precursor concentration. Size of the MBN NPs, as determined by DLS, as a function of concentration of Fe^{II}. The concentration of PA was also varied, while maintaining the molar ratio of Fe^{II}-to-PA at 1:1. Data are presented as the mean ± SD, $n = 3$.

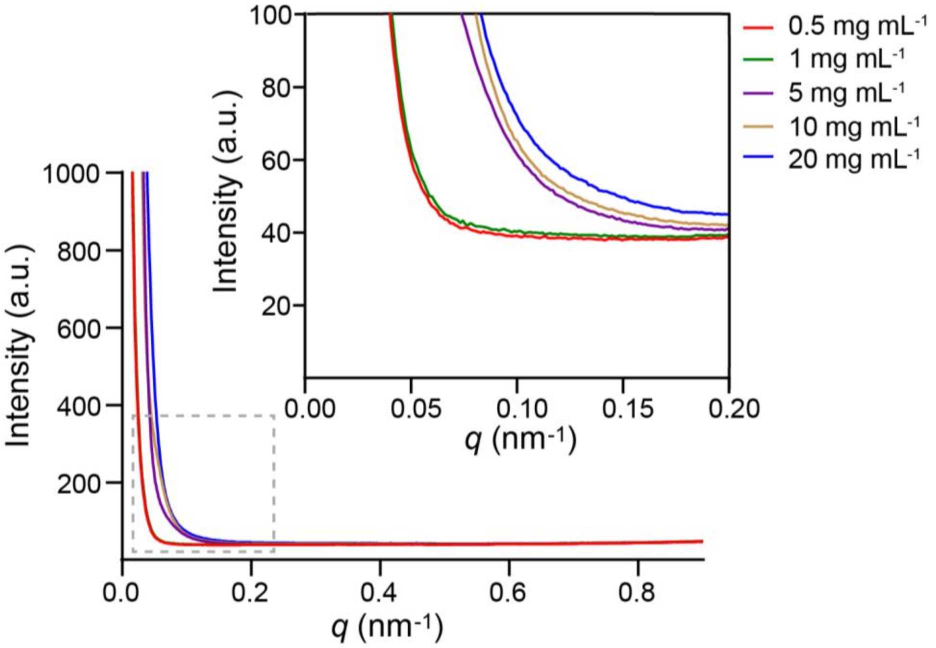

Fig. S6. SAXS characterization of MBN NPs. SAXS patterns of MBN NPs assembled using different concentrations of Fe^{II}. The molar ratio of Fe^{II}-to-PA was maintained at 1:1.

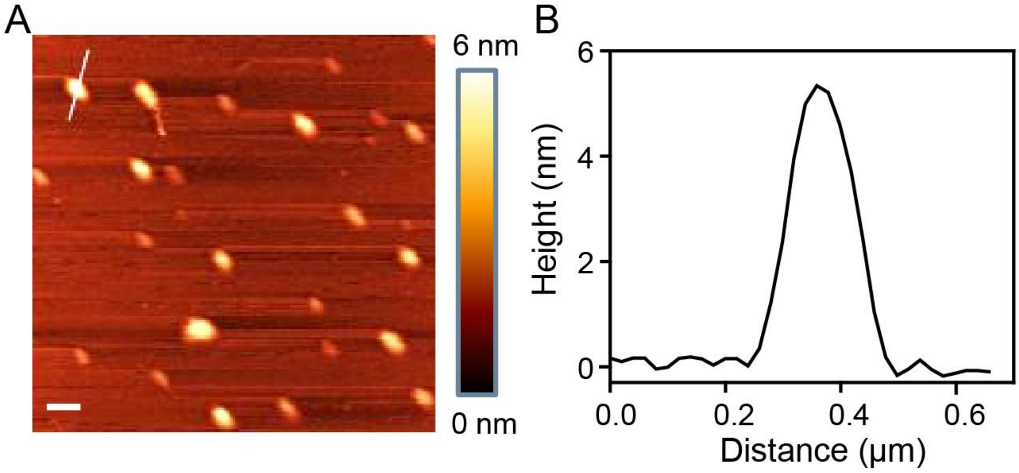

Fig. S7. AFM characterization of MBN NPs. (A) AFM image and (B) corresponding thickness profile of MBN (Fe^{II}–PA) NPs. Scale bar is 200 nm.

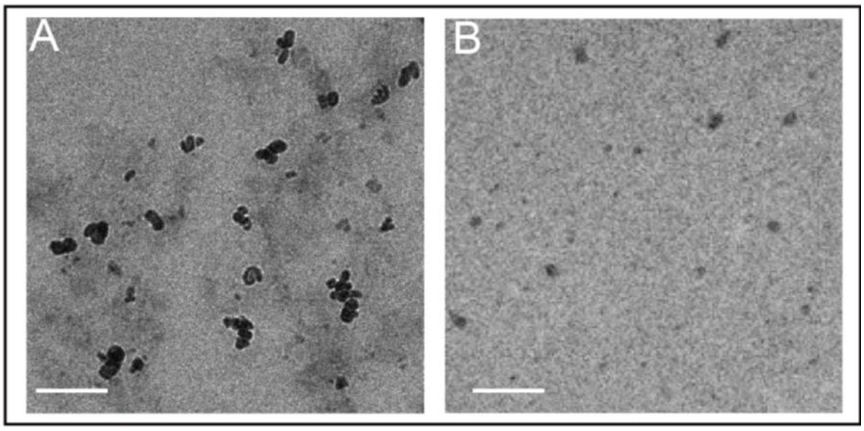

Fig. S8. TEM characterization of MBN NPs. TEM images of (A) Fe^{II}–PA and (B) Zr^{IV}–PA NPs. Scale bars are 200 nm.

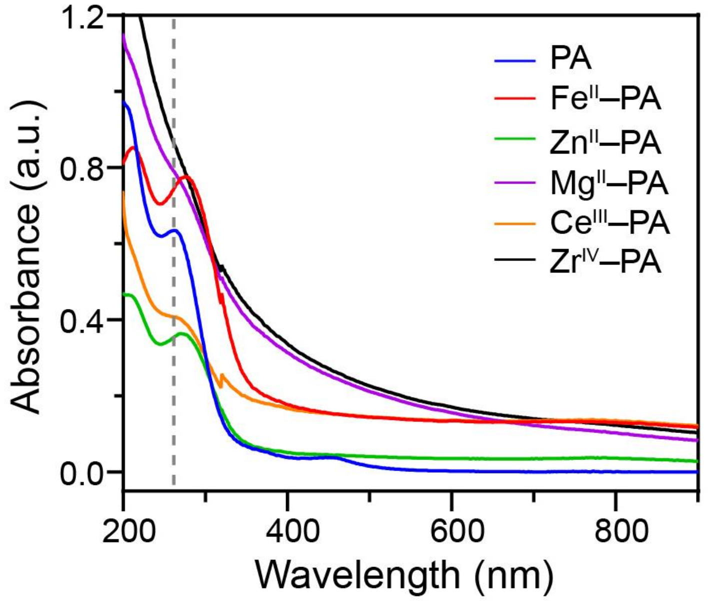

Fig. S9. UV–vis spectra of PA, and MBN NPs prepared with PA and different metal ions. The dashed line indicates the characteristic absorbance band of PA at 260 nm.

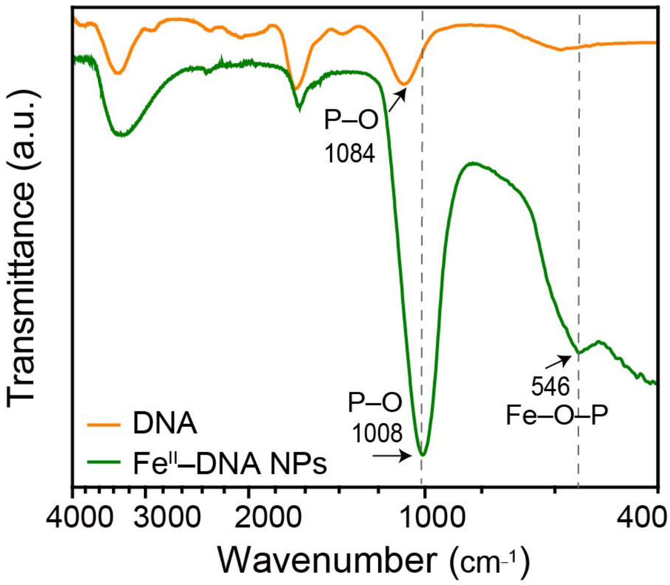

Fig. S10. FTIR spectra of DNA and Fe^{II}–DNA NPs. $cDNA_{18}$ was used for sample preparation.

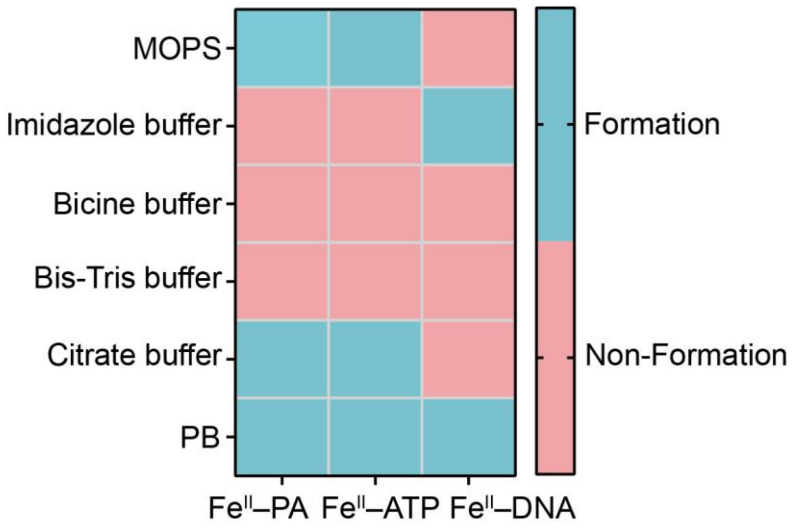

Fig. S11. Effect of buffer solution on MBN NP formation. Summary of successful and unsuccessful formation of MBN NPs (Fe^{II}–PA, Fe^{II}–ATP, and Fe^{II}–DNA NPs) in various buffers at pH 7. The formation of nanosized NPs was assessed by DLS.

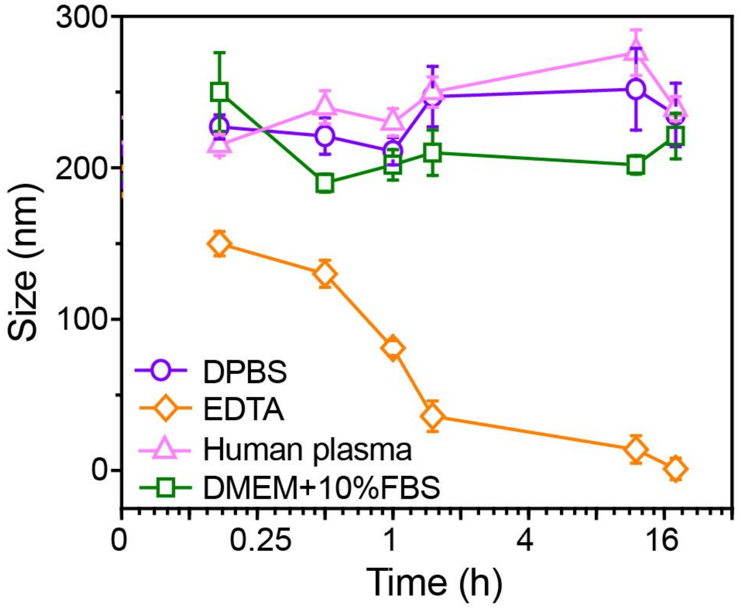

Fig. S12. Stability of MBN NPs upon incubation in different solvents. Stability of MBN (Fe^{II}–PA) NPs upon incubation in EDTA and different culture media (DPBS, human plasma, and DMEM+10%FBS), as assessed by changes in NP size by DLS. Data are presented as the mean ± SD, *n* = 3.

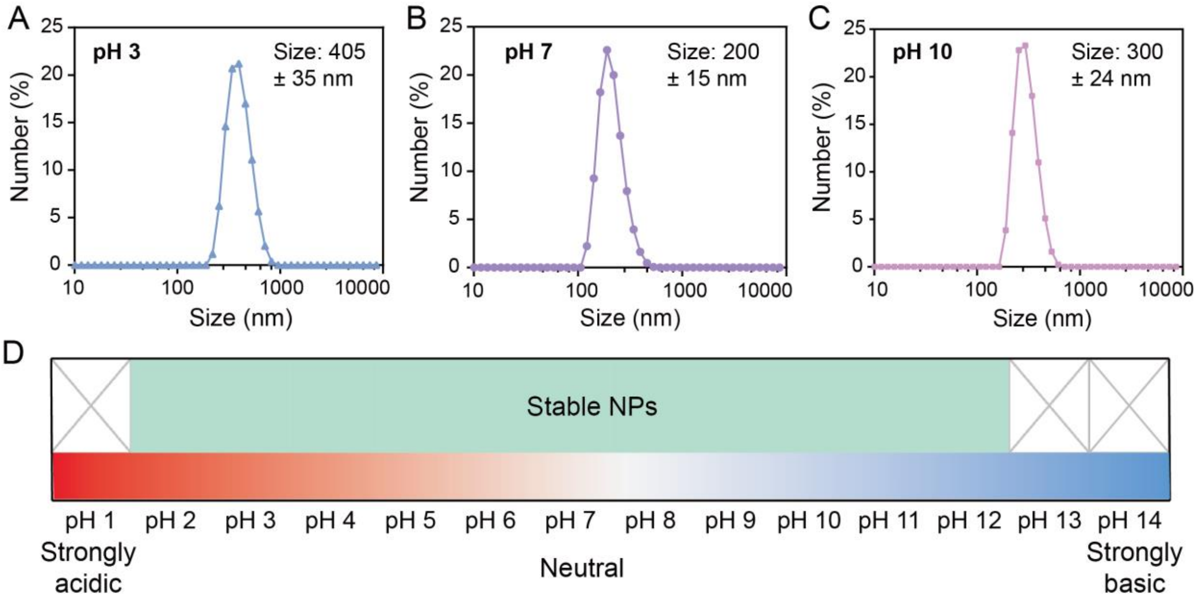

Fig. S13. Stability of MBN NPs upon incubation in different pH solutions. (A–C) DLS data of MBN (Fe^{II}–PA) NPs upon incubation in different pH solutions. (D) Heatmap summarizing the pH stability of MBN (Fe^{II}–PA) NPs.

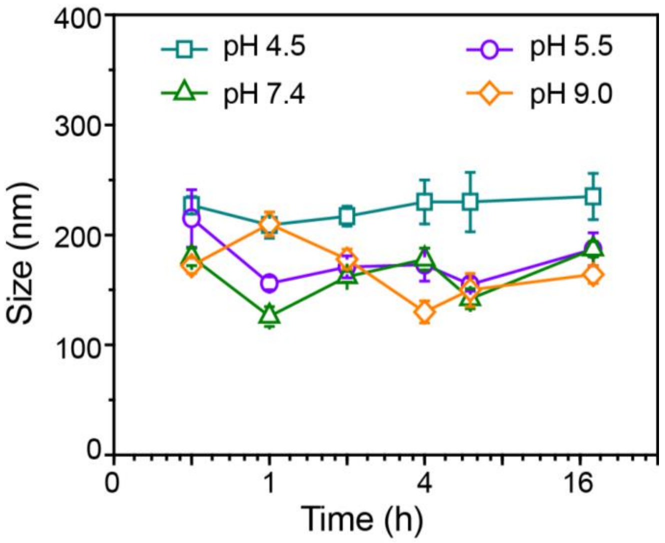

Fig. S14. Stability of Fe^{II}–DNA NPs upon incubation in different pH solutions. Data are shown as the mean ± SD (*n* = 3).

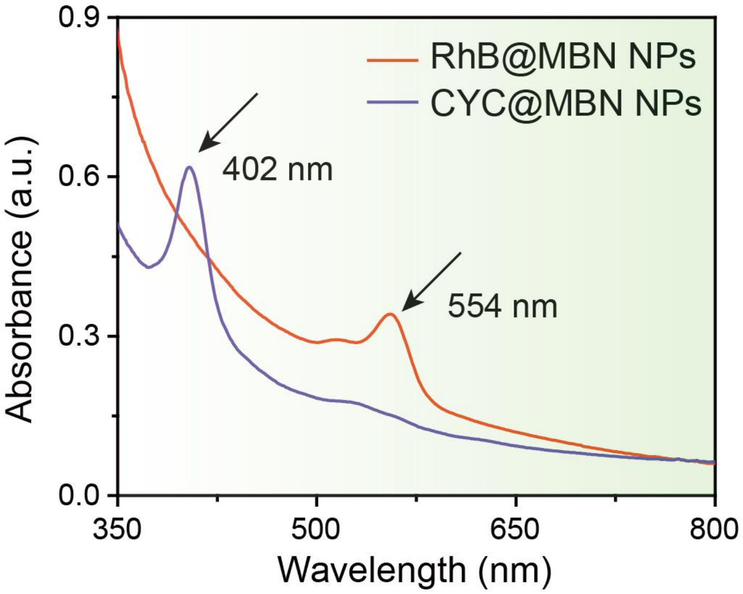

Fig. S15. UV–vis spectra of RhB@MBN NPs and CYC@MBN NPs. The characteristic peaks of RhB and CYC at 554 and 402 nm, respectively, are indicated. Fe^{II} and PA were used as the NP building blocks.

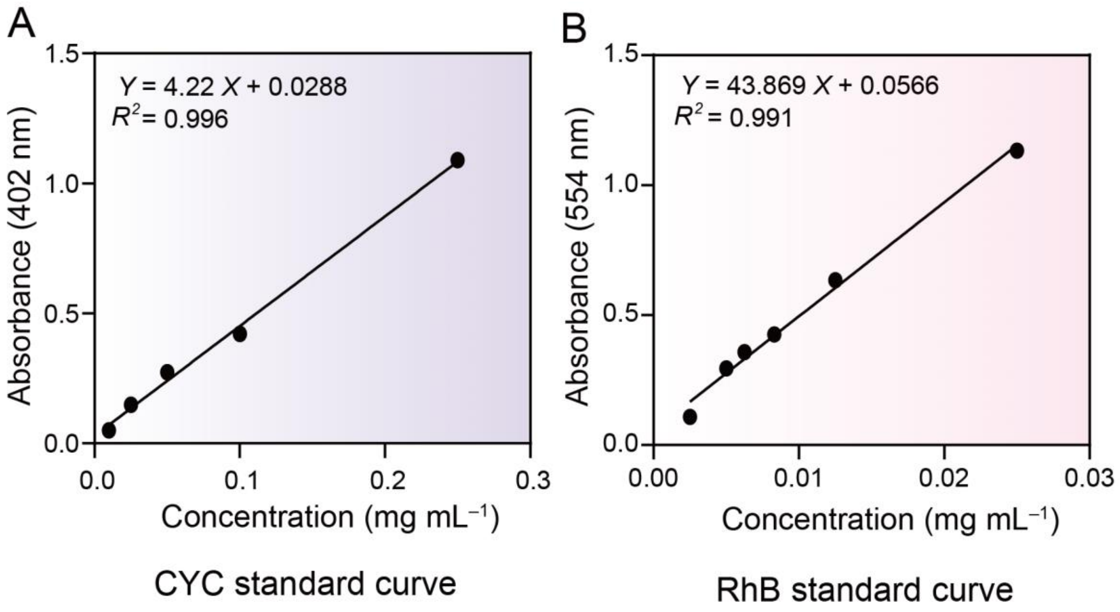

Fig. S16. Concentration standard curves of different proteins. Standard curves of (A) CYC concentration measured at 402 nm and (B) RhB concentration measured at 554 nm, as established by UV–vis spectrophotometry.

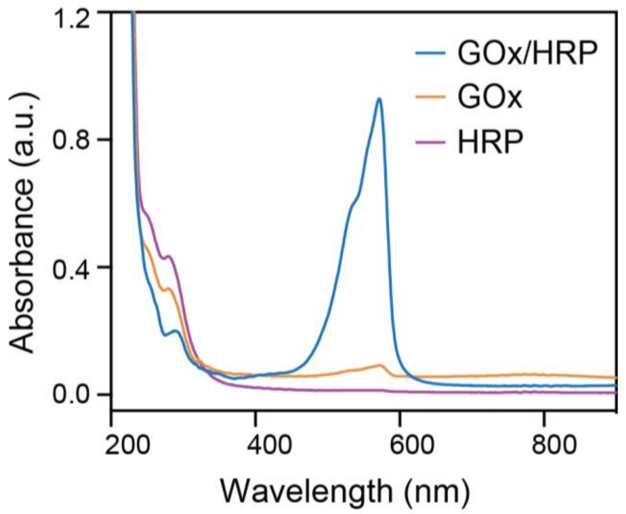

Fig. S17. Enzymatic cascade reaction of enzyme-loaded MBN NPs. UV–vis spectra of the cascade reaction using different single-component (GOx@MBN NPs or HRP@MBN NPs) and multicomponent (GOx/HRP@MBN NPs) NPs. MBN NPs were formed using Fe^{II} and PA NP building blocks.

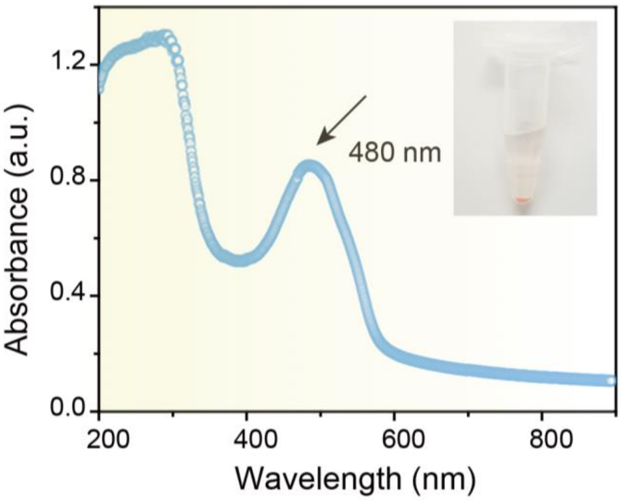

Fig. S18. DOX-loaded MBN NPs. UV–vis spectra of DOX@MBN (Fe^{II}–PA) NPs and photograph (inset) of the NPs (colored pellets) following centrifugation of the NP dispersion.

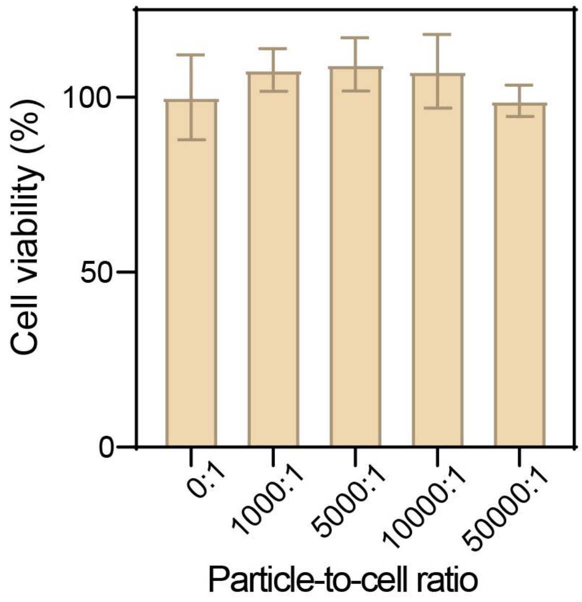

Fig. S19. Cytotoxicity of MBN NPs toward HeLa cells. Viability of HeLa cells after incubation with MBN (Fe^{II}–PA) NPs at different particle-to-cell ratios. Data are presented as the mean ± SD (n = 5).

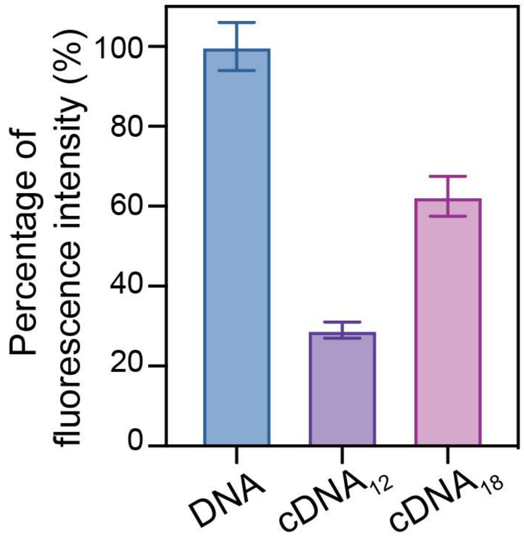

Fig. S20. Complementary binding or hybridization of metal–DNA NPs. Changes in the fluorescence intensity of fluorescein amidate-labeled DNA strands in the Fe^{II}–DNA NPs upon addition of Cy3-labeled $cDNA_{12}$ and $cDNA_{18}$. Data are presented as the mean ± SD, $n = 3$.

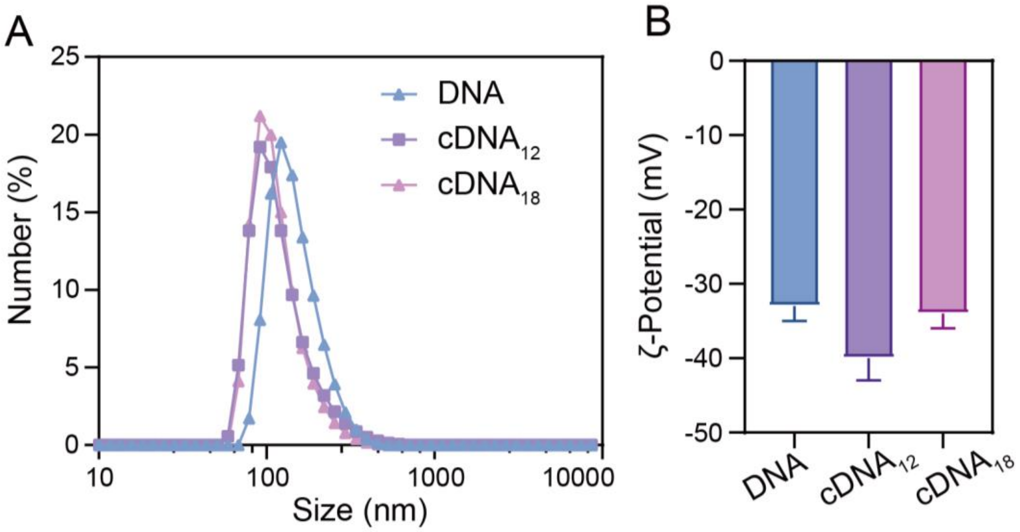

Fig. S21. Molecular recognition of MBP NPs. (A) DLS data and (B) ζ-potential values of Fe^{II}–DNA NPs upon hybridization and displacement with Cy3-labeled $cDNA_{12}$ and $cDNA_{18}$, respectively. Data are presented as the mean ± SD, $n = 3$.

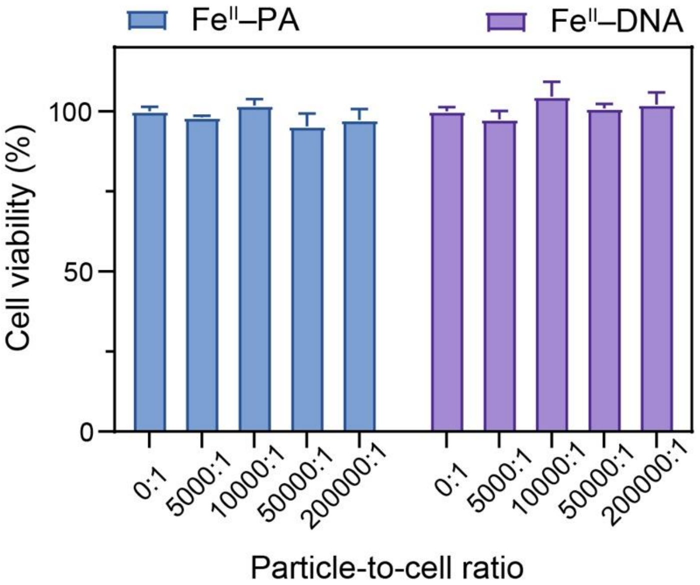

Fig. S22. Cytotoxicity of MPB NPs toward HEK 293T cells. Viability of HEK 293T cells after incubation with Fe^{II}–PA NPs or Fe^{II}–DNA NPs at different particle-to-cell ratios. Data are presented as the mean ± SD, n = 5.

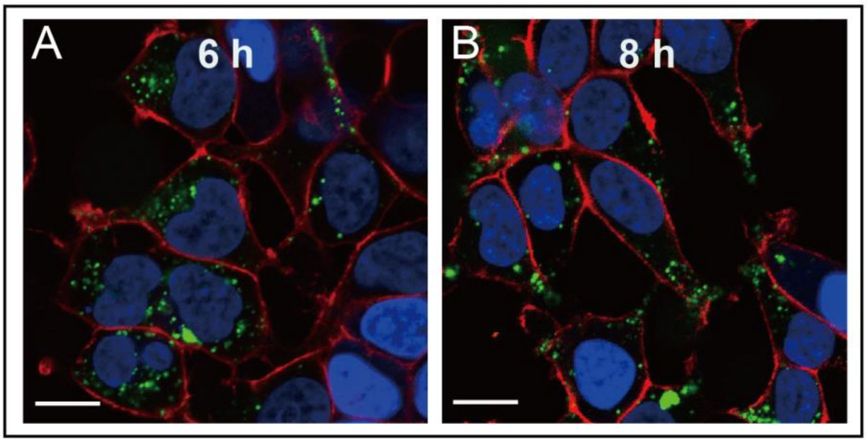

Fig. S23. Cell internalization of MBP NPs. CLSM images showing the internalization of FITC-BSA@MBN NPs by HEK 293T cells after incubation for (A) 6 and (B) 8 h. Green, FITC-BSA@MBN NPs; blue, nuclei; and red, cell membrane. Scale bars are 10 μm. The MFP NPs were prepared using Fe^{II} and PA.

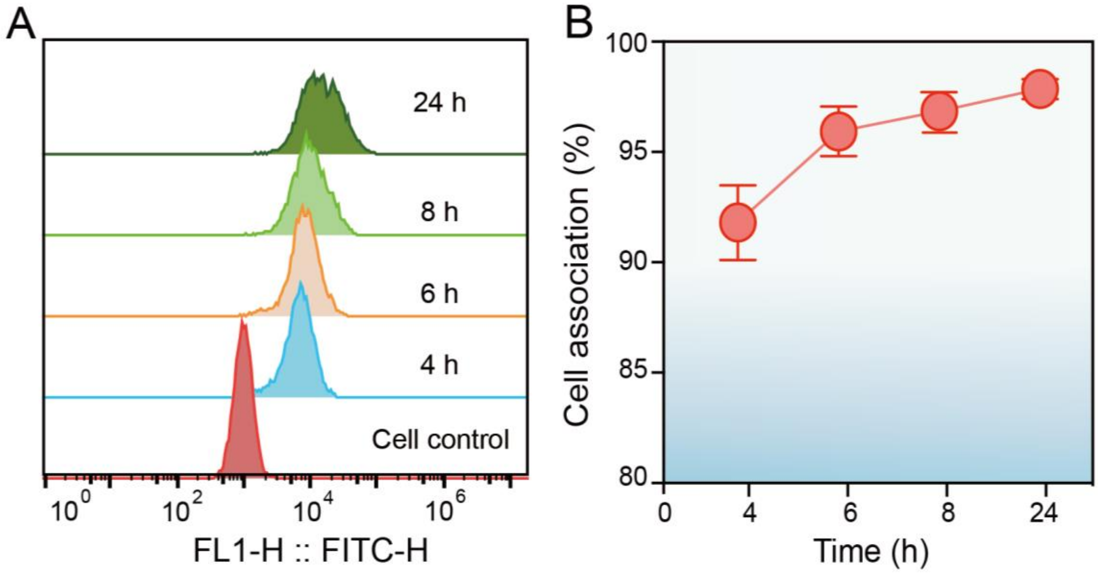

Fig. S24. Cell association of MBN NPs. (A) Histogram of FITC fluorescence of HEK 293T cells following incubation with FITC-BSA@MBN NPs for 4, 6, 8, and 24 h. (B) Time-dependent association of the FITC-BSA@MPF NPs with HEK 293T cells. The MFP NPs were prepared using Fe^{II} and PA.

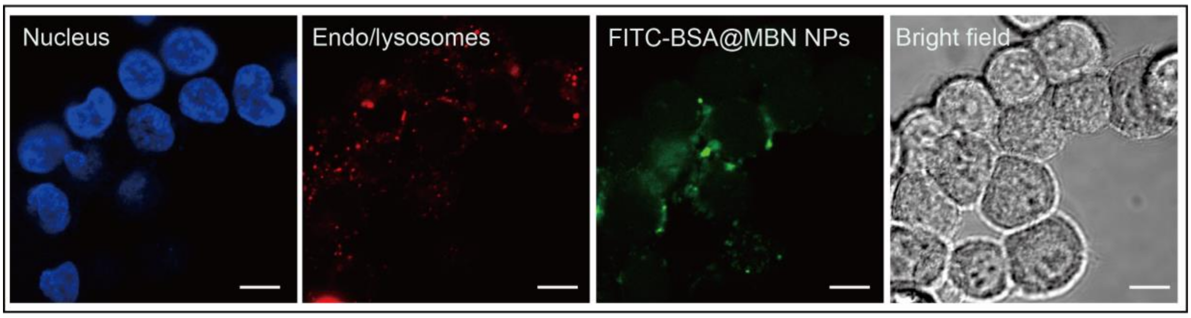

Fig. S25. Endosomal escape of MBN NPs. CLSM images of HEK 293T cells incubated with FITC-BSA@MBN NPs for 8 h at a particle-to-cell ratio of 50000:1. Green, FITC-BSA@MBN NPs; red, endosomes and lysosomes; blue, nuclei. Scale bars are 10 μm. The MBN NPs were prepared using Fe^{II} and PA.

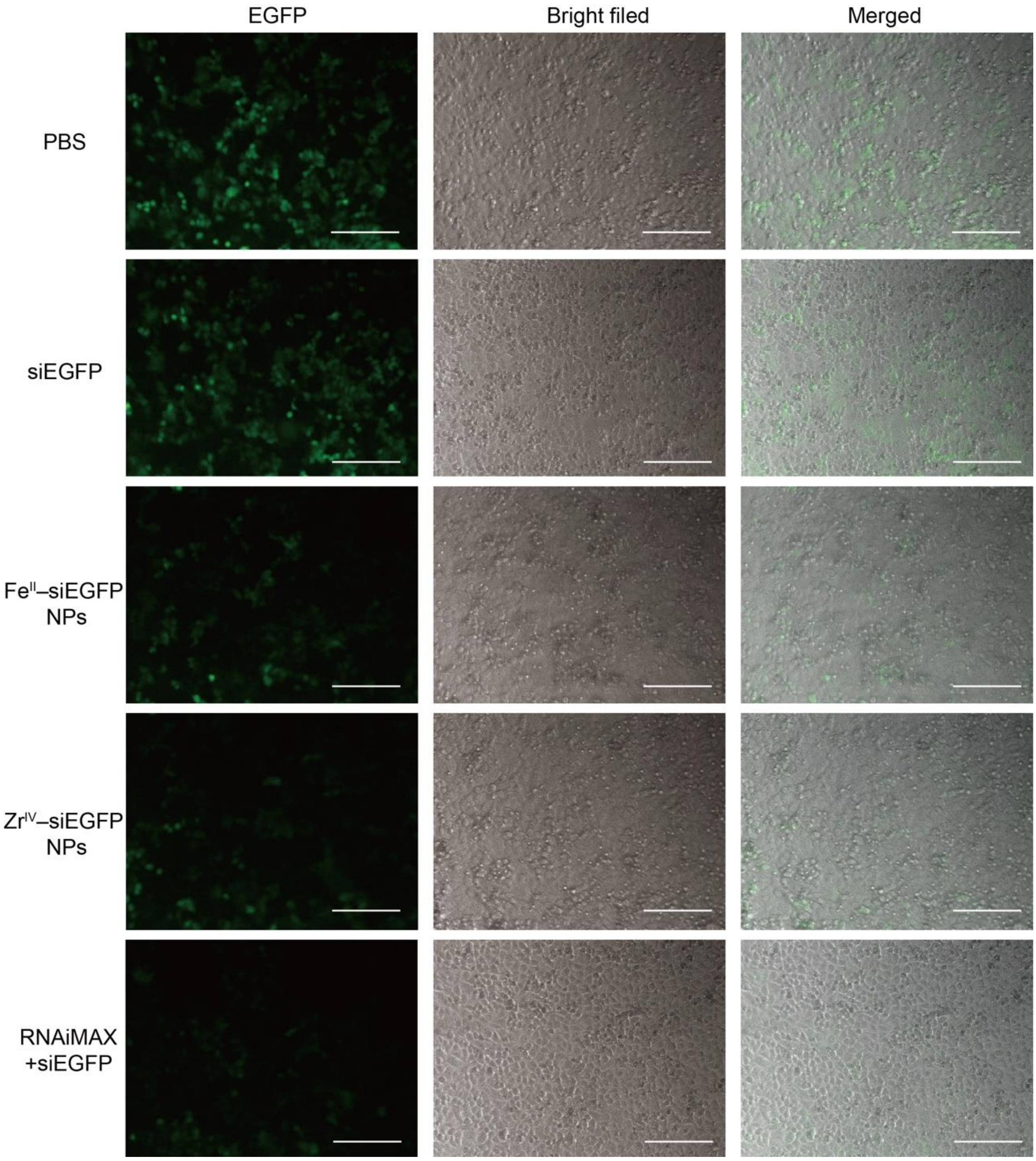

Fig. S26. Metal–siEGFP NP-mediated EGFP overexpression silencing. Fluorescence images of HeLa–EGFP cells treated with siEGFP-containing formulations and showing the silencing of EGFP expression. Scale bars: 100 μm.

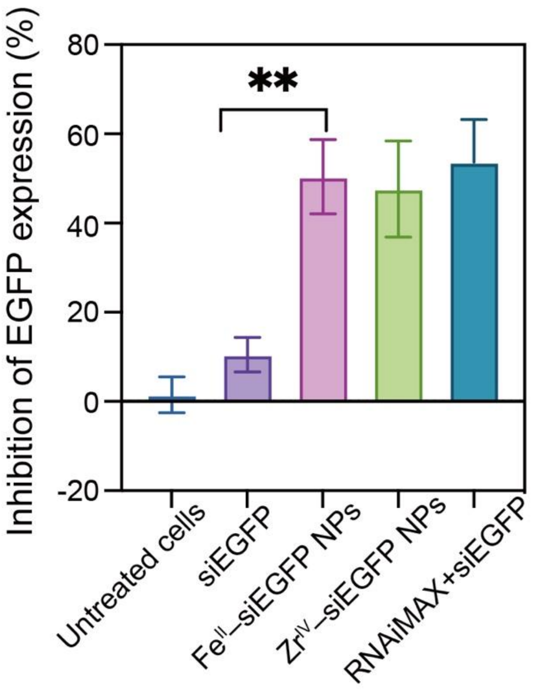

Fig. S27. Gene knockdown of metal–siEGFP NPs. Inhibition of EGFP expression from HeLa–EGFP cells after incubation with different siEGFP-containing formulations for 48 h. Data are shown as the mean ± SD (n = 3). t-test: **P < 0.01.

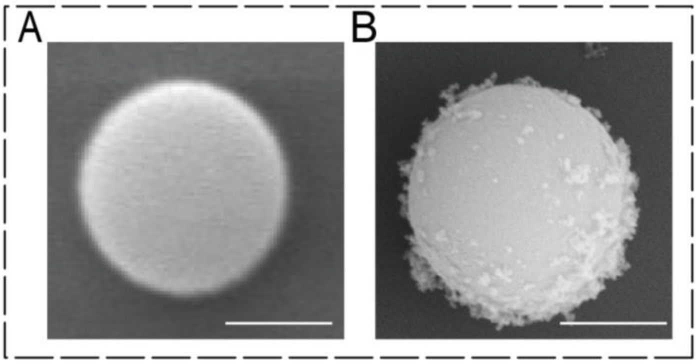

Fig. S28. SEM imaging of a representative MPN supraparticle formed on a particle template. Representative SEM images of (A) a PS-NH_2 template particle and (B) an MBN supraparticle. The supraparticle was prepared from interactions between Fe^{II}–DNA NPs and Fe^{II}–$cDNA_{18}$ NPs on a PS-NH_2 template particle. Scale bars are 1 μm.

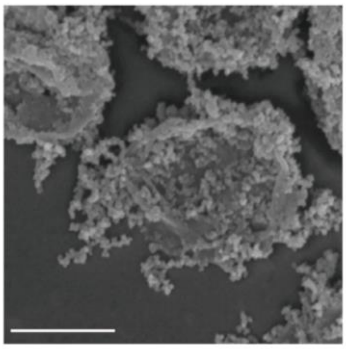

Fig. S29. SEM image of a representative air-dried hollow supraparticle. Supraparticles were first constructed from interactions between Fe^{II}–DNA NPs and Fe^{II}–$cDNA_{18}$ NPs on PS-NH_2 template particles. Hollow supraparticles were then obtained following removal of the template particles. Scale bar is 1 μm.

Section S2. Supporting Tables

Table S1. DNA sequences

**Name[a)]**	**Sequence**
FAM-labeled DNA	5′-A3-ATCCTTATCAATATTCAA-FAM-3′
Cy3-labled $cDNA_{12}$	5′-Cy3-TTGAATATTGAT-3′
$cDNA_{18}$	5′-TTGAATATTGATAAGGAT-3′
Cy3-labled $cDNA_{18}$	5′- Cy3-TTGAATATTGATAAGGAT-3′
CpG	GGTTACCACCTTCATTGGAAAACGTTCTTCGGGGCGTTCTTAGGTGG TAACC

[a)] DNA and cDNA are complementary.

Table S2. Hybridization binding energy with DNA1(FAM) at 25 °C generated using NUPACK[a)]

	Hybridization binding energy [kcal mol^{-1}]
Cy3-labled $cDNA_{12}$	–11.29
$cDNA_{18}$	–19.70

a) http://www.nupack.org/.

Checklist

Minimum Information Reporting in Bio–Nano Experimental Literature

The MIRIBEL guidelines were introduced here: https://doi.org/10.1038/s41565-018-0246-4

The development of these guidelines was led by the ARC Centre of Excellence in Convergent Bio-Nano Science and Technology: https://www.cbns.org.au/. Any updates or revisions to this document will be made available here: http://doi.org/10.17605/OSF.IO/SMVTF. This document is made available under a CC-BY 4.0 license: https://creativecommons.org/licenses/by/4.0/.

The MIRIBEL guidelines were developed to facilitate reporting and dissemination of research in bio–nano science. Their development was inspired by various similar efforts:

- MIAME (microarray experiments): *Nat. Genet.* **29** (2001), 365; http://doi.org/10.1038/ng1201-365
- MIRIAM (biochemical models): *Nat. Biotechnol.* **23** (2005) 1509; http://doi.org/10.1038/nbt1156
- MIBBI (biology/biomedicine): *Nat. Biotechnol.* **26** (2008) 889; http://doi.org/10.1038/nbt.1411
- MIGS (genome sequencing): *Nat. Biotechnol.* **26** (2008) 541; http://doi.org/10.1038/nbt1360
- MIQE (quantitative PCR): *Clin. Chem.* **55** (2009) 611; http://doi.org/10.1373/clinchem.2008.112797
- ARRIVE (animal research): *PLOS Biol.* **8** (2010) e1000412; http://doi.org/10.1371/journal.pbio.1000412
- *Nature*'s reporting standards:
    - Life science: https://www.nature.com/authors/policies/reporting.pdf; e.g., *Nat. Nanotechnol.* **9** (2014) 949; http://doi.org/10.1038/nnano.2014.287
    - Solar cells: https://www.nature.com/authors/policies/solarchecklist.pdf; e.g., *Nat. Photonics* **9** (2015) 703; http://doi.org/10.1038/nphoton.2015.233
    - Lasers: https://www.nature.com/authors/policies/laserchecklist.pdf; e.g., *Nat. Photonics* **11** (2017) 139; http://doi.org/10.1038/nphoton.2017.28
- The "TOP guidelines": e.g., *Science* **352** (2016) 1147; http://doi.org/10.1126/science.aag2359

Similar to many of the efforts listed above, the parameters included in this checklist are **not** intended to be definitive requirements; instead they are intended as 'points to be considered', with authors themselves deciding which parameters are—and which are not—appropriate for their specific study.

This document is intended to be a living document, which we propose is revisited and amended annually by interested members of the community, who are encouraged to contact the authors of this document. Parts of this document were developed at the annual International Nanomedicine Conference in Sydney, Australia: http://www.oznanomed.org/, which will continue to act as a venue for their review and development, and interested members of the community are encouraged to attend.

After filling out the following pages, this checklist document can be attached as a "Supporting Information" document during submission of a manuscript to inform Editors and Reviewers (and eventually readers) that all points of MIRIBEL have been considered.

Supplementary Table 1. Material characterization*

**Question**	**Yes**	**No**
1.1 Are "**best reporting practices**" **available** for the nanomaterial used? For examples, see *Chem. Mater*. **28** (2016) 3535; http://doi.org/10.1021/acs.chemmater.6b01854 and *Chem. Mater*. **29** (2017) 1; http://doi.org/10.1021/acs.chemmater.6b05235	**Not applicable**	
1.2 If they are available, **are they used**? If not available, ignore this question and proceed to the next one.		
1.3 Are extensive and clear instructions reported detailing all steps of **synthesis** and the resulting **composition** of the nanomaterial? For examples, see *Chem. Mater*. **26** (2014) 1765; http://doi.org/10.1021/cm500632c, and *Chem. Mater*. **26** (2014) 2211; http://doi.org/10.1021/cm5010449. Extensive use of photos, images, and videos are strongly encouraged. For example, see *Chem. Mater*. **28** (2016) 8441; http://doi.org/10.1021/acs.chemmater.6b04639	√	
1.4 Is the **size** (or **dimensions**, if non-spherical) and **shape of** the nanomaterial reported?	√	
1.5 Is the **size dispersity** or **aggregation** of the nanomaterial reported?	√	
1.6 Is the **zeta potential** of the nanomaterial reported?	√	
1.7 Is the **density (mass/volume)** of the nanomaterial reported?	**Not applicable**	
1.8 Is the amount of any **drug loaded** reported? 'Drug' here broadly refers to functional cargos (e.g., proteins, small molecules, nucleic acids).	√	
1.9 Is the **targeting performance** of the nanomaterial reported, including **amount** of ligand bound to the nanomaterial if the material has been functionalised through addition of targeting ligands?	**Not applicable**	
1.10 Is the **label signal** per nanomaterial/particle reported? For example, fluorescence signal per particle for fluorescently labelled nanomaterials.	√	
1.11 If a material property not listed here is varied, has it been **quantified**?	√	
1.12 Were characterizations performed in a **fluid mimicking biological conditions**?	**Not applicable**	
1.13 Are details of how these parameters were **measured/estimated** provided?	√	
Explanation for **No** (if needed):		

***Ideally, material characterization should be performed in the same biological environment as that in which the study will be conducted. For example, for cell culture studies with nanoparticles, characterization steps would ideally be performed on nanoparticles dispersed in cell culture media. If this is not possible, then characteristics of the dispersant used (e.g., pH, ionic strength) should mimic as much as possible the biological environment being studied.

Supplementary Table 2. Biological characterization*

Question	Yes	No
2.1 Are **cell seeding details**, including **number of cells plated, confluency at start of experiment,** and **time between seeding and experiment** reported?	√	
2.2 If a standardised cell line is used, are the **designation and source** provided?	√	
2.3 Is the **passage number** (total number of times a cell culture has been subcultured) known and reported?	**Not applicable**	
2.4 Is the last instance of **verification of cell line** reported? If no verification has been performed, is the time passed and passage number since acquisition from trusted source (e.g., ATCC or ECACC) reported? For information, see *Science* **347** (2015) 938; http://doi.org/10.1126/science.347.6225.938	**Not applicable**	
2.5 Are the results from **mycoplasma testing** of cell cultures reported?	**Not applicable**	
2.6 Is the **background signal of cells/tissue** reported? (E.g., the fluorescence signal of cells without particles in the case of a flow cytometry experiment.)	√	
2.7 Are **toxicity studies** provided to demonstrate that the material has the expected toxicity, and that the experimental protocol followed does not?	√	
2.8 Are details of media preparation (**type of media, serum,** any **added antibiotics**) provided?	√	
2.9 Is a **justification of the biological model** used provided? For examples for cancer models, see *Cancer Res.* **75** (2015) 4016; http://doi.org/10.1158/0008-5472.CAN-15-1558, and *Mol. Ther.* **20** (2012) 882; http://doi.org/10.1038/mt.2012.73, and *ACS Nano* **11** (2017) 9594; http://doi.org/10.1021/acsnano.7b04855	√	
2.10 Is characterization of the **biological fluid** (*ex vivo*/*in vitro*) reported? For example, when investigating protein adsorption onto nanoparticles dispersed in blood serum, pertinent aspects of the blood serum should be characterised (e.g., protein concentrations and differences between donors used in study).	**Not applicable**	
2.11 For **animal experiments**, are the ARRIVE guidelines followed? For details, see *PLOS Biol.* **8** (2010) e1000412; http://doi.org/10.1371/journal.pbio.1000412	**Not applicable**	
Explanation for **No** (if needed):		

*For *in vitro* experiments (e.g., cell culture), *ex vivo* experiments (e.g., in blood samples), and *in vivo* experiments (e.g., animal models). The questions above that are appropriate depend on the type of experiment conducted.

Supplementary Table 3. Experimental details*

Question	Yes	No
3.1 For cell culture experiments: are **cell culture dimensions** including **type of well**, **volume of added media**, reported? Are cell types (i.e.; adherent vs suspension) and **orientation** (if non-standard) reported?	√	
3.2 Is the **dose of material administered** reported? This is typically provided in nanomaterial mass, volume, number, or surface area added. Is sufficient information reported so that regardless of which one is provided, the other dosage metrics can be calculated (i.e. using the dimensions and density of the nanomaterial)?	√	
3.3 For each type of imaging performed, are details of how **imaging** was performed provided, including details of **shielding, non-uniform image processing**, and any **contrast agents** added?	√	
3.4 Are details of how the dose was administered provided, including **method of administration, injection location, rate of administration,** and details of **multiple injections**?	**Not applicable**	
3.5 Is the methodology used to **equalise dosage** provided?	√	
3.6 Is the **delivered dose** to tissues and/or organs (in vivo) reported, as % injected dose per gram of tissue (%ID g^{-1})?	**Not applicable**	
3.7 Is **mass of each organ/tissue measured** and **mass of material** reported?	**Not applicable**	
3.8 Are the **signals of cells/tissues with nanomaterials** reported? For instance, for fluorescently labelled nanoparticles, the total number of particles per cell or the fluorescence intensity of particles + cells, at each assessed timepoint.	√	
3.9 Are **data analysis details**, including **code used** for analysis provided?	√	
3.10 Is the **raw data** or **distribution of values** underlying the reported results provided? For examples, see *R. Soc. Open Sci.* **3** (2016) 150547; http://doi.org/10.1098/rsos.150547, https://opennessinitiative.org/making-your-data-public/, http://journals.plos.org/plosone/s/data-availability, and https://www.nature.com/sdata/policies/repositories	√	
Explanation for **No** (if needed):		

* The use of protocol repositories (e.g., *Protocol Exchange* http://www.nature.com/protocolexchange/) and published standard methods and protocols (e.g., *Chem. Mater.* **29** (2017) 1; http://doi.org/10.1021/acs.chemmater.6b05235, and *Chem. Mater.* **29** (2017) 475; http://doi.org/10.1021/acs.chemmater.6b05481) are encouraged.

REFERENCES AND NOTES

1. H. Li, M. Eddaoudi, M. O'Keeffe, O. M. Yaghi, Design and synthesis of an exceptionally stable and highly porous metal–organic framework. *Nature* **402**, 276–279 (1999).

2. H. Furukawa, K. E. Cordova, M. O'Keeffe, O. M. Yaghi, The chemistry and applications of metal-organic frameworks. *Science* **341**, 1230444 (2013).

3. S. Dang, Q.-L. Zhu, Q. Xu, Nanomaterials derived from metal–organic frameworks. *Nat. Rev. Mater.* **3**, 17075 (2018).

4. G. Chakraborty, I.-H. Park, R. Medishetty, J. J. Vittal, Two-dimensional metal-organic framework materials: Synthesis, structures, properties and applications. *Chem. Rev.* **121**, 3751–3891 (2021).

5. X. Zhang, X. Tian, N. Wu, S. Zhao, Y. Qin, F. Pan, S. Yue, X. Ma, J. Qiao, W. Xu, W. Liu, J. Liu, M. Zhao, K. Ostrikov, Z. Zeng, Metal–organic frameworks with fine-tuned interlayer spacing for microwave absorption. *Sci. Adv.* **10**, eadl6498 (2024).

6. H. Robatjazi, D. Weinberg, D. F. Swearer, C. Jacobson, M. Zhang, S. Tian, L. Zhou, P. Nordlander, N. J. Halas, Metal–organic frameworks tailor the properties of aluminum nanocrystals. *Sci. Adv.* **5**, eaav5340 (2019).

7. A. Knebel, J. Caro, Metal–organic frameworks and covalent organic frameworks as disruptive membrane materials for energy-efficient gas separation. *Nat. Nanotechnol.***17**, 911–923 (2022).

8. J. Liu, D. Xie, X. Xu, L. Jiang, R. Si, W. Shi, P. Cheng, Reversible formation of coordination bonds in Sn-based metal–organic frameworks for high-performance lithium storage. *Nat. Commun.* **12**, 3131 (2021).

9. H. Ejima, J. J. Richardson, K. Liang, J. P. Best, M. P. van Koeverden, G. K. Such, J. Cui, F. Caruso, One-step assembly of coordination complexes for versatile film and particle engineering. *Science* **341**, 154–157 (2013).

10. H. Geng, Q.-Z. Zhong, J. Li, Z. Lin, J. Cui, F. Caruso, J. Hao, Metal ion-directed functional metal–phenolic materials. *Chem. Rev.* **122**, 11432–11473 (2022).

11. W. Xu, S. Pan, B. B. Noble, J. Chen, Z. Lin, Y. Han, J. Zhou, J. J. Richardson, I. Yarovsky, F. Caruso, Site-selective coordination assembly of dynamic metal–phenolic networks. *Angew. Chem. Int. Ed. Engl.* **61**, e202208037 (2022).

12. J. Zhou, Z. Lin, Y. Ju, M. A. Rahim, J. J. Richardson, F. Caruso, Polyphenol-mediated assembly for particle engineering. *Acc. Chem. Res.* **53**, 1269–1278 (2020).

13. W. Xu, S. Pan, B. B. Noble, Z. Lin, S. K. Bhangu, C.-J. Kim, J. Chen, Y. Han, I. Yarovsky, F. Caruso, Engineering flexible metal–phenolic networks with guest responsiveness via intermolecular interactions. *Angew. Chem. Int. Ed. Engl.* **62**, e202302448 (2023).

14. Z. Guo, T. Liu, W. Gao, C. Iffelsberger, B. Kong, M. Pumera, Multi-wavelength light-responsive metal–phenolic network-based microrobots for reactive species scavenging. *Adv. Mater.* **35**, 2210994 (2023).

15. G. Lin, J. J. Richardson, H. Ahmed, Q. A. Besford, A. J. Christofferson, S. Beyer, Z. Lin, A. R. Rezk, M. Savioli, J. Zhou, C. F. McConville, C. Cortez-Jugo, L. Y. Yeo, F. Caruso, Programmable phototaxis of metal–phenolic particle microswimmers. *Adv. Mater.* **33**, 2006177 (2021).

16. Z. Wang, J. Gao, J. Zhou, J. Gong, L. Shang, H. Ye, F. He, S. Peng, Z. Lin, Y. Li, F. Caruso, Engineering metal–phenolic networks for solar desalination with directional salt crystallization. *Adv. Mater.* **35**, 2209015 (2023).

17. J. Su, P. Wang, W. Zhou, M. Peydayesh, J. Zhou, T. Jin, F. Donat, C. Jin, L. Xia, K. Wang, F. Ren, P. Van der Meeren, F. P. G. de Arquer, R. Mezzenga, Single-site iron-anchored amyloid hydrogels as catalytic platforms for alcohol detoxification. *Nat. Nanotechnol.* **19**, 1168–1177 (2024).

18. Z. Lin, J. J. Richardson, J. Zhou, F. Caruso, Direct synthesis of amorphous coordination polymers and metal–organic frameworks. *Nat. Rev. Chem.* **7**, 273–286 (2023).

19. L. Moretto, M. Ušaj, O. Matusovsky, D. E. Rassier, R. Friedman, A. Månsson, Multistep orthophosphate release tunes actomyosin energy transduction. *Nat. Commun.* **13**, 4575 (2022).

20. N. Chen, N. Du, R. Shen, T. He, J. Xi, J. Tan, G. Bian, Y. Yang, T. Liu, W. Tan, L. Yu, Q. Yuan, Redox signaling-driven modulation of microbial biosynthesis and biocatalysis. *Nat. Commun.* **14**, 6800 (2023).

21. K. Tighanimine, J. A. N. L. F. Freitas, I. Nemazanyy, A. Bankolé, D. Benarroch-Popivker, S. Brodesser, G. Doré, L. Robinson, P. Benit, S. Ladraa, Y. B. Saada, B. Friguet, P. Bertolino, D. Bernard, G. Canaud, P. Rustin, E. Gilson, O. Bischof, S. Fumagalli, M. Pende, A homoeostatic switch causing glycerol-3-phosphate and phosphoethanolamine accumulation triggers senescence by rewiring lipid metabolism. *Nat. Metab.* **6**, 323–342 (2024).

22. S. K. Aulakh, S. J. Varma, M. Ralser, Metal ion availability and homeostasis as drivers of metabolic evolution and enzyme function. *Curr. Opin. Genet. Dev.* **77**, 101987 (2022).

23. J. Wieruszewska, A. Pawłowicz, E. Połomska, K. Pasternak, Z. Gdaniec, W. Andrałojć, The 8-17 DNAzyme can operate in a single active structure regardless of metal ion cofactor. *Nat. Commun.* **15**, 4218 (2024).

24. X. Zeng, T. Wei, X. Wang, Y. Liu, Z. Tan, Y. Zhang, T. Feng, Y. Cheng, F. Wang, B. Ma, W. Qin, C. Gao, J. Xiao, C. Wang, Discovery of metal-binding proteins by thermal proteome profiling. *Nat. Chem. Biol.* **20**, 770–778 (2024).

25. F. Li, Z. Lv, X. Zhang, Y. Dong, X. Ding, Z. Li, S. Li, C. Yao, D. Yang, Supramolecular self-assembled DNA nanosystem for synergistic chemical and gene regulations on cancer cells. *Angew. Chem. Int. Ed. Engl.* **60**, 25557–25566 (2021).

26. W. Ma, Y. Zhan, Y. Zhang, C. Mao, X. Xie, Y. Lin, The biological applications of DNA nanomaterials: Current challenges and future directions. *Signal Transduct. Target. Ther.* **6**, 351 (2021).

27. S. Wang, C. M. McGuirk, A. d'Aquino, J. A. Mason, C. A. Mirkin, Metal–organic framework nanoparticles. *Adv. Mater.* **30**, 1800202 (2018).

28. R. Ettlinger, U. Lächelt, R. Gref, P. Horcajada, T. Lammers, C. Serre, P. Couvreur, R. E. Morris, S. Wuttke, Toxicity of metal–organic framework nanoparticles: From essential analyses to potential applications. *Chem. Soc. Rev.* **51**, 464–484 (2022).

29. W. Xu, Z. Lin, S. Pan, J. Chen, T. Wang, C. Cortez-Jugo, F. Caruso, Direct assembly of metal–phenolic network nanoparticles for biomedical applications. *Angew. Chem. Int. Ed. Engl.* **62**, e202312925 (2023).

30. J. Chen, S. Pan, J. Zhou, Z. Lin, Y. Qu, A. Glab, Y. Han, J. J. Richardson, F. Caruso, Assembly of bioactive nanoparticles via metal–phenolic complexation. *Adv. Mater.* **34**, 2108624 (2022).

31. S. Aqdim, M. Ouchetto, Elaboration and structural investigation of iron (III) phosphate glasses. *Adv. Mater. Phys. Chem.* **3**, 332–339 (2013).

32. L. D. Carli, E. Schnitzler, M. Ionashiro, B. Szpoganicz, N. D. Rosso, Equilibrium, thermoanalytical and spectroscopic studies to characterize phytic acid complexes with Mn (II) and Co (II). *J. Braz. Chem. Soc.* **20**, 1515–1522 (2009).

33. S. M. D. Watson, H. D. A. Mohamed, B. R. Horrocks, A. Houlton, Electrically conductive magnetic nanowires using an electrochemical DNA-templating route. *Nanoscale* **5**, 5349–5359 (2013).

34. D. Luna-Zaragoza, E. T. Romero-Guzmán, L. R. Reyes Gutiérrez, Surface and physicochemical characterization of phosphates vivianite, $Fe_2(PO_4)_3$ and hydroxyapatite, $Ca_5(PO_4)_3OH$. *J. Miner. Mater. Charact. Eng.* **8**, 591–609 (2009).

35. T. Zheng, Z. Yang, D. Gui, Z. Liu, X. Wang, X. Dai, S. Liu, L. Zhang, Y. Gao, L. Chen, D. Sheng, Y. Wang, J. Diwu, J. Wang, R. Zhou, Z. Chai, T. E. Albrecht-Schmitt, S. Wang, Overcoming the crystallization and designability issues in the ultrastable zirconium phosphonate framework system. *Nat. Commun.* **8** 15369 (2017).

36. Y. Zhang, S. Tsitkov, H. Hess, Proximity does not contribute to activity enhancement in the glucose oxidase–horseradish peroxidase cascade. *Nat. Commun.* **7**, 13982 (2016).

37. M. Hu, C. Feng, Q. Yuan, C. Liu, B. Ge, F. Sun, X. Zhu, Lantern-shaped flexible RNA origami for *Smad4* mRNA delivery and growth suppression of colorectal cancer. *Nat. Commun.* **14**, 1307 (2023).

38. S. Oehler, L. Lucaroni, F. Migliorini, A. Elsayed, L. Prati, S. Puglioli, M. Matasci, K. Schira, J. Scheuermann, D. Yudin, M. Jia, N. Ban, D. Bushnell, R. Kornberg, S. Cazzamalli, D. Neri, N. Favalli, G. Bassi, A DNA-encoded chemical library based on chiral 4-amino-proline enables stereospecific isozyme-selective protein recognition. *Nat. Chem.***15**, 1431–1443 (2023).

39. Q. Mou, X. Xue, Y. Ma, M. Banik, V. Garcia, W. Guo, J. Wang, T. Song, L.-Q. Chen, Y. Lu, Efficient delivery of a DNA aptamer-based biosensor into plant cells for glucose sensing through thiol-mediated uptake. *Sci. Adv.* **8**, eabo0902 (2022).

40. S.-J. Park, T. A. Taton, C. A. Mirkin, Array-based electrical detection of DNA with nanoparticle probes. *Science* **295**, 1503–1506 (2002).

41. X. Zhou, H. Pu, D.-W. Sun, DNA functionalized metal and metal oxide nanoparticles: Principles and recent advances in food safety detection. *Crit. Rev. Food Sci. Nutr.* **61**, 2277–2296 (2021).

42. J. Chen, J. Li, J. Zhou, Z. Lin, F. Cavalieri, E. Czuba-Wojnilowicz, Y. Hu, A. Glab, Y. Ju, J. J. Richardson, F. Caruso, Metal–phenolic coatings as a platform to trigger endosomal escape of nanoparticles. *ACS Nano*, **13**, 11653–1664 (2019).

43. T. He, J. Wen, W. Wang, Z. Hu, C. Ling, Z. Zhao, Y. Cheng, Y.-C. Chang, M. Xu, Z. Jin, L. Amer, L. Sasi, L. Fu, N. F. Steinmetz, T. M. Rana, P. Wu, J. V Jokerst, Peptide-driven proton sponge nano-assembly for imaging and triggering lysosome-regulated immunogenic cancer cell death. *Adv. Mater.* **36**, 2307679 (2024).

44. D. M. Klinman, Immunotherapeutic uses of CpG oligodeoxynucleotides. *Nat. Rev. Immunol.* **4**, 249–259 (2004).

45. Q. Sun, M. Barz, B. G. De Geest, M. Diken, W. E. Hennink, F. Kiessling, T. Lammers, Y. Shi, Nanomedicine and macroscale materials in immuno-oncology. *Chem. Soc. Rev.* **48**, 351–381 (2019).

46. C. Volpi, F. Fallarino, M. T. Pallotta, R. Bianchi, C. Vacca, M. L. Belladonna, C. Orabona, A. De Luca, L. Boon, L. Romani, U. Grohmann, P. Puccetti, High doses of CpG oligodeoxynucleotides stimulate a tolerogenic TLR9–TRIF pathway. *Nat. Commun.* **4**, 1852 (2013).

47. K. Portmann, A. Linder, N. Oelgarth, K. Eyer, Single-cell deep phenotyping of cytokine release unmasks stimulation-specific biological signatures and distinct secretion dynamics. *Cell Rep. Methods* **3**, 100502 (2023).

48. G. Altan-Bonnet, R. Mukherjee, Cytokine-mediated communication: A quantitative appraisal of immune complexity. *Nat. Rev. Immunol.* **19**, 205–217 (2019).

49. B. Hu, L. Zhong, Y. Weng, L. Peng, Y. Huang, Y. Zhao, X. J. Liang, Therapeutic siRNA: State of the art. *Signal Transduct. Target. Ther.* **5**, 101 (2020).

50. M. Xiao, W. Lai, T. Man, B. Chang, L. Li, A. R. Chandrasekaran, H. Pei, Rationally engineered nucleic acid architectures for biosensing applications. *Chem. Rev.* **119**, 11631–11717 (2019).

51. L. Li, H. Xing, J. Zhang, Y. Lu, Functional DNA molecules enable selective and stimuli-responsive nanoparticles for biomedical applications. *Acc. Chem. Res.* **52**, 2415–2426 (2019).

52. J. Zhang, Z. Di, H. Yan, Y. Zhao, L. Li, One-step synthesis of single-stranded DNA-bridged iron oxide supraparticles as MRI contrast agents. *Nano Lett.* **21**, 2793–2799 (2021).

53. S. Wang, S. S. Park, C. T. Buru, H. Lin, P.-C. Chen, E. W. Roth, O. K. Farha, C. A. Mirkin, Colloidal crystal engineering with metal–organic framework nanoparticles and DNA. *Nat. Commun.* **11**, 2495 (2020).

54. J. Guo, B. L. Tardy, A. J. Christofferson, Y. Dai, J. J. Richardson, W. Zhu, M. Hu, Y. Ju, J. Cui, R. R. Dagastine, I. Yarovsky, F. Caruso, Modular assembly of superstructures from polyphenol-functionalized building blocks. *Nat. Nanotechnol.* **11**, 1105–1111 (2016).

55. Z. Lin, H. Liu, J. J. Richardson, W. Xu, J. Chen, J. Zhou, F. Caruso, Metal–phenolic network composites: From fundamentals to applications. *Chem. Soc. Rev.* **53**, 10800–10826 (2024).

56. M. Faria, M. Björnmalm, K. J. Thurecht, S. J. Kent, R. G. Parton, M. Kavallaris, A. P. R. Johnston, J. J. Gooding, S. R. Corrie, B. J. Boyd, P. Thordarson, A. K. Whittaker, M. M. Stevens, C. A. Prestidge, C. J. H. Porter, W. J. Parak, T. P. Davis, E. J. Crampin, F. Caruso, Minimum information reporting in bio-nano experimental literature. *Nat. Nanotechnol.* **13**, 777–785 (2018).

57. D. S. Marynick, H. F. Schaefer III, Theoretical studies of metal-phosphate interactions: Interaction of Li^+, Na^+, K^+, Be^{++}, Mg^{++}, and Ca^{++} with $H_2PO_4^-$ and $(CH_3O)_2PO_2^-$: Implications for nucleic acid solvation. *Proc. Natl. Acad. Sci. U.S.A.* **72**, 3794–3798 (1975).

58. B. Schneider, M. Kabeláč, P. Hobza, Geometry of the phosphate group and its interactions with metal cations in crystals and *ab initio* calculations. *J. Am. Chem. Soc.* **118**, 12207–12217 (1996).
